# Which Factors Affect the Occurrence of Off-Target Effects Caused by the Use of CRISPR/Cas: A Systematic Review in Plants

**DOI:** 10.3389/fpls.2020.574959

**Published:** 2020-11-23

**Authors:** Dominik Modrzejewski, Frank Hartung, Heike Lehnert, Thorben Sprink, Christian Kohl, Jens Keilwagen, Ralf Wilhelm

**Affiliations:** Federal Research Centre for Cultivated Plants, Institute for Biosafety in Plant Biotechnology, Julius Kühn-Institute, Quedlinburg, Germany

**Keywords:** genome editing, unintended effects, off-target mutation, systematic review and meta-analysis, systematic literature search, evidence synthesis, new plant breeding technique

## Abstract

CRISPR/Cas enables a targeted modification of DNA sequences. Despite their ease and efficient use, one limitation is the potential occurrence of associated off-target effects. This systematic review aims to answer the following research question: Which factors affect the occurrence of off-target effects caused by the use of CRISPR/Cas in plants? Literature published until March 2019 was considered for this review. Articles were screened for relevance based on pre-defined inclusion criteria. Relevant studies were subject to critical appraisal. All studies included in the systematic review were synthesized in a narrative report, but studies rated as *high* and *medium/high validity* were reported separately from studies rated as *low* and *medium/low* or *unclear validity*. In addition, we ran a binary logistic regression analysis to verify five factors that may affect the occurrence of off-target effects: (1) Number of mismatches (2) Position of mismatches (3) GC-content of the targeting sequence (4) Altered nuclease variants (5) Delivery methods. In total, 180 relevant articles were included in this review containing 468 studies therein. Seventy nine percentage of these studies were rated as having *high* or *medium/high validity*. Within these studies, 6,416 potential off-target sequences were assessed for the occurrence of off-target effects. Results clearly indicate that an increased number of mismatches between the on-target and potential off-target sequence steeply decreases the likelihood of off-target effects. The observed rate of off-target effects decreased from 59% when there is one mismatch between the on-target and off-target sequences toward 0% when four or more mismatches exist. In addition, mismatch/es located within the first eight nucleotides proximal to the PAM significantly decreased the occurrence of off-target effects. There is no evidence that the GC-content significantly affects off-target effects. The database regarding the impact of the nuclease variant and the delivery method is very poor as the majority of studies applied the standard nuclease SpCas9 and the CRISPR/Cas system was stably delivered in the genome. Hence, a general significant impact of these two factors on the occurrence of off-target effects cannot be proved. This identified evidence gap needs to be filled by systematic studies exploring these individual factors in sufficient numbers.

## Background

Site-directed nucleases (SDN), including Clustered Regularly Interspaced Short Palindromic Repeats with associated protein (e.g., CRISPR/Cas9), Transcription Activator-Like Effector Nucleases (TALENs), Zinc-Finger Nucleases (ZFN) and Meganucleases (MN) enable a modification of a specific DNA sequences in a site-directed manner. Compared to other SDN, CRISPR/Cas is the most widely applied method due to simplicity, accessibility, lower costs and versatility as well as the possibility of multiplexing (Dönmez et al., 2016). Most CRISPR/Cas systems require two elements for targeted genome cleavage: An endonuclease and a single guide RNA (sgRNA) which recognizes and binds to a specific DNA sequence that is typically 20 base pairs long for SpCas9 (Sprink et al., 2016). The to date most commonly used endonuclease Cas9 derived from *Streptococcus pyogenes* is able to bind to any DNA sequence paired with an sgRNA followed by a protospacer adjacent motif (PAM) site (NGG for *S. pyogenes*) and introduces a double strand break (DSB) three bases upstream of the PAM (Zhu et al., 2017). The DSB is subsequently repaired by the DNA repair system of the cell. CRISPR/Cas has already been applied all over the world in more than 60 plant species ranging from model organisms to agricultural crops (Modrzejewski et al., 2019). Despite the obvious potentials of CRISPR/Cas in plant breeding, there is an ongoing debate about its precise targeting and to what extent the occurrence of off-target effects matters (Eckerstorfer et al., 2019b, Zhao and Wolt, 2017). In order to evaluate potential side effects and to further enhance the specificity of CRISPR/Cas system, a detailed evaluation on the occurrence of off-target effects is important (Martin et al., 2016). Off-target effects can be defined as unintended cleavage and mutations at untargeted genomic sites showing a similar but not an identical sequence compared to the target site (Modrzejewski et al., 2019). It is not exactly known why the Cas9 protein cleaves some off-target sites and others not. One important factor affecting cleavage of Cas9 could be chromatin structure as it has been shown that Cas9 cleaves more efficient in open chromatin regions (Hinz et al., 2015; Liu et al., 2019). In addition, a number of biophysical experiments highlighted that not only PAM proximal but also PAM distal mismatches can strongly inhibit off-target cleavage. The proposed mechanism for this intriguing phenomenon is a balance shift in the HNH domain toward an inactive state (Chen et al., 2017; Dagdas et al., 2017; Ricci et al., 2019; Mitchell et al., 2020). After binding of the target DNA and establishment of the RNA:DNA hybrid at the on-target, the HNH domain flips from an RNA-bound state to an inactive state and then rapidly into a docked-state that enables cleavage (Dagdas et al., 2017). PAM distal mismatches between the sgRNA and the DNA (especially position 16–20) can slow down or even inhibit this transition from inactive to docked-state, thereby prohibiting off-target cleavage (Chen et al., 2017; Dagdas et al., 2017). This biophysical results lead to the conclusion, that not only RNA:DNA binding affects the occurrence of off-target cleavage but also the internal reorganization of the HNH domain. The first effect seems to be located PAM proximal as we could show also in this review by analysis of a number of experimentally validated off-target effects. The second effect is located PAM distal and does not rely on RNA:DNA binding. In contrast to this, mismatches in the more upstream positions 15–10 do almost not affect the cleavage negatively, as they neither have a remarkable effect on RNA:DNA duplex stability nor on the HNH conformational changes (Chen et al., 2017).

Two different strategies have been used to detect off-target effects: biased and unbiased detection methods (Martin et al., 2016). To date, the majority of studies analyzed off-target effects using biased detection methods (Modrzejewski et al., 2019). This approach consists of two steps. First, the potential off-target sequences that are similar to the target sequence are identified using bioinformatics approaches like CAS-OFF-Finder (Altschul, 1990), CRISPR-P (Lei et al., 2014; Liu et al., 2017), CHOPCHOP (Montague et al., 2014) or CCTop (Stemmer et al., 2015). Second, the identified potential off-target sequences only are analyzed for undesired mutations (off-target effects). Several different detection methods are used, whereby off-target effects were mostly analyzed using PCR followed by sequencing (Modrzejewski et al., 2019). Nevertheless, a drawback of solely screening pre-selected potential off-target sequences is the risk to overlook mutations at further loci in the plant genome (Zischewski et al., 2017). In contrast, using the unbiased approach whole genome sequencing (WGS) is the most common detection method in plants allowing the identification of off-target effects in a less restricted way (Martin et al., 2016; Modrzejewski et al., 2019). However, due to the high costs today, solely a few clones are sequenced for each target in most instances, and hence low-frequency off-target effects might be missed because of low coverage (read depth) (Wu et al., 2014). Mutations detected by using unbiased detection methods do not necessarily originate from the application of a genome editing technique, but may also result from spontaneous mutations or through the regeneration of plants from cell culture (somaclonal variation) (Scientific Advice Mechanism (SAM), 2017). Therefore, a suitable reference genome is needed to detect genetic differences. In order to trace these differences to a genome editing alteration, bioinformatics and statistical analyses are necessary to estimate whether these differences are consisterably likely to be technology-induced genetic modifications (Bartsch et al., 2018). Compared to routinely used breeding techniques like undirected mutagenesis (e.g., by irradiation or chemicals) or somaclonal variation, CRISPR/Cas causes far less unintended changes (Jander et al., 2003; Miyao et al., 2012; Scientific Advice Mechanism (SAM), 2017). Nevertheless, a recently published systematic map on off-target effects in CRISPR/Cas-studies identified several publications, which reported the identification of off-target effects (Modrzejewski et al., 2019). It is estimated that monomeric CRISPR/Cas system is more prone for the occurrence of off-target effects compared to the dimeric ZFN or TALENs systems that use two neighboring target sequences and hence recognize a longer target sequence (Lee et al., 2016; Zischewski et al., 2017). In addition, the sgRNA can tolerate some mismatches and bulges and hence efforts have been made to increase the on-target efficiency and to decrease the occurrence of off-target effects.

Several factors are described in literature that may affect off-target effects. The main factor to decrease the occurrence of off-target effects is a careful selection of the target sequence (Hsu et al., 2013; Zhu et al., 2017; Zischewski et al., 2017). Design tools like CRISPR-P or CHOPCHOP provide the possibility to take potential off-target sequences into account when choosing the target sequence (Zhao and Wolt, 2017). It was considered that mismatches occurring within the seed sequence (8 up to 12 nucleotides proximal to the PAM) determine the editing efficiency. Therefore, mismatches within this region may reduce off-target effects (Hsu et al., 2013; Endo et al., 2015; Hahn and Nekrasov, 2018). However, it is not clear how many nucleotides proximal to the PAM are building up the seed sequence. Different researchers define the seed sequence to be eight (Russo et al., 2018), nine (Bertier et al., 2018), 10 (Jiang et al., 2017), 11 (Jacobs et al., 2015), or up to 12 (LeBlanc et al., 2018) nucleotides proximal to the PAM.

Scientists also assume that the guanine-cytosine (GC) content of a chosen target sequence may influence off-target effects, as high GC-content stabilize the sRNA/genomic DNA hybridization (Fu et al., 2013). Yu et al. (2017) speculate that a low GC-content results in an reduced number of off-target effects (Yu et al., 2017). Russo et al. (2018) have chosen a target sequence with a GC-content of 50% as they assume that a GC-content higher than 70% may increase the risk of off-targeting (Russo et al., 2018).

Much effort is invested in examining other nucleases and developing improved ones. Next to the most widely used nuclease SpCas9 derived from *Streptococcus pyogenes* further Cas9 proteins from different bacterial or archaea species have been adapted. One example is the Cas9 derived from *Staphylococcus aureus* (SaCas9) that is capable to use a longer recognition sequence of 21- or 22- nucleotides and a different PAM (NNGRRT). Therefore fewer off-target sequences are predicted *per se* and its specificity is estimated to be higher (Kaya et al., 2016). In addition, the Cpf1 nuclease (also known as Cas12a) has been applied for targeted genome modification in plants. Compared to Cas9, the Cpf1 (from *Prevoltella sp*. and *Francisella sp*.) recognizes a T-rich PAM (TTTV) at the 5'end instead of the 3'end of the protospacer and has the potential to decrease off-target effects due to their DNA recognition and cutting properties (Hahn and Nekrasov, 2018). In addition, protein-engineering approaches resulted in altered SpCas9 variants with potentially reduced off-target activity. Several SpCas9-variants like SpCas9-HF, eSpCas9 (1.0), or eSpCas9 (1.1) have been engineered and were already applied in plants (Zhang et al., 2017; Raitskin et al., 2019). These nuclease variants were described to nearly entirely avoid off-targeting (Tycko et al., 2016). Another promising approach is the application of CRISPR/nickase systems. In contrast to nucleases which induce a DSB a nickase system can be used to induce single strand breaks or as a paired nickase system to induce offset DSBs (Puchta and Fauser, 2014; Zhao and Wolt, 2017). This may reduce off-target effects as the recognition site is doubled from 20 to 40 nucleotides (Puchta and Fauser, 2014).

A further aspect that may have an impact on off-target effects is the delivery of the CRISPR/Cas system into the plant cell. The system can be supplied to the plant genome either as DNA (transiently or stably expressed), RNA or directly as Ribonucleoproteins (RNP) (Jansing et al., 2019). It is supposed that the occurrence of off-target effects depends on how long the CRISPR/Cas system is active in the plant cell (Jansing et al., 2019). Stable transformation leads to a permanent expression of the CRISPR/Cas system compared to the transient approach in which the CRISPR/Cas system is available only for a limited time. Therefore, it is supposed that a stable transformation leads to an increased on-target as well as an increased off-target activity (Zischewski et al., 2017; Metje-Sprink et al., 2018; Jansing et al., 2019). Supplying the CRISPR/Cas system as RNA or RNP may further reduce off-target effects, as it is degraded in the shortest period and the mode of action is only present in the edited cells but not in the regenerated plants (Woo et al., 2015; Metje-Sprink et al., 2018; Jansing et al., 2019). All delivery methods have their specific advantages and disadvantages and are more or less suitable for different plant species (Jansing et al., 2019).

All in all a broad range of factors may affect the occurrence of off-target effects. Reviewing all available literature for plants, to date no systematical analyzes, which factors actually affect the occurrence of off-target effects due to the application of CRISPR/Cas in plants were found.

### Topic Identification

Risk assessors and decision makers are depending on the provision of a reliable body of evidence to support conclusions about potential risks being associated with the application of genome editing. In this context, the (potential) off-target effects caused by genome editing in contrast to its broadly claimed precision are a point of lasting criticism as they might lead to adverse alterations in plants (Eckerstorfer et al., 2019a). Additionally, the detailed analysis of the occurrence of off-target effects can support further enhancement of the specificity of CRISPR/Cas (Martin et al., 2016). This systematic review builds on the recently published systematic map on genome editing applications in plants (Modrzejewski et al., 2019). The systematic map and the a priori published systematic map protocol were conducted based on the guideline of Collaboration for Environmental Evidence (CEE) aiming to take the reader through the key stages of the review (Collaboration for Environmental Evidence, 2018). One topic of this map (secondary question 2) was the identification of the available evidence for the occurrence of such off-target effects. The map identified a knowledge cluster of publications considering the evaluation of off-target effects caused by CRISPR/Cas in plants (other genome editing tools were much less represented), supporting the conduct of an in depth analysis by a systematic review on this specific section of the map. In the reviewed studies, factors potentially modifying the occurrence of off-target effects such as the plant species, the nuclease-variant [e.g., Cas9, Cpf1(Cas12a)], the number of mismatches and the chosen sgRNA were mapped (Modrzejewski et al., 2019). Based on these data, this systematic review identifies and systematically analyzes factors that may affect the occurrence of off-target effects caused by CRISPR/Cas in plants.

## Objectives of the Review

The primary objective of this systematic review is to collect and synthesize the available evidence about factors that do affect the occurrence of off-target effects caused by the application of CRISPR/Cas in plants.

Primary question: “Which factors affect the occurrence of off-target effects caused by the use of CRISPR/Cas in plants”?

### Components of the Primary Question

*Population*: Any model plant or crop produced for agricultural production.

*Intervention*: The CRISPR/Cas technique was used to induce any on-target mutation.

*Outcome*: The occurrence of off-target effects was assessed. Either biased or unbiased detection methods were used to check whether off-target effects occurred in the plant genome.

## Methods

The methods used to conduct this systematic review are based on the recently published systematic map (Modrzejewski et al., 2019), but adapted to the specific requirements of the systematic review question. The methods specifically used to conduct this systematic review as well as the deviations compared to the systematic map protocol are described below.

### Search for Articles

**Figure 1:**
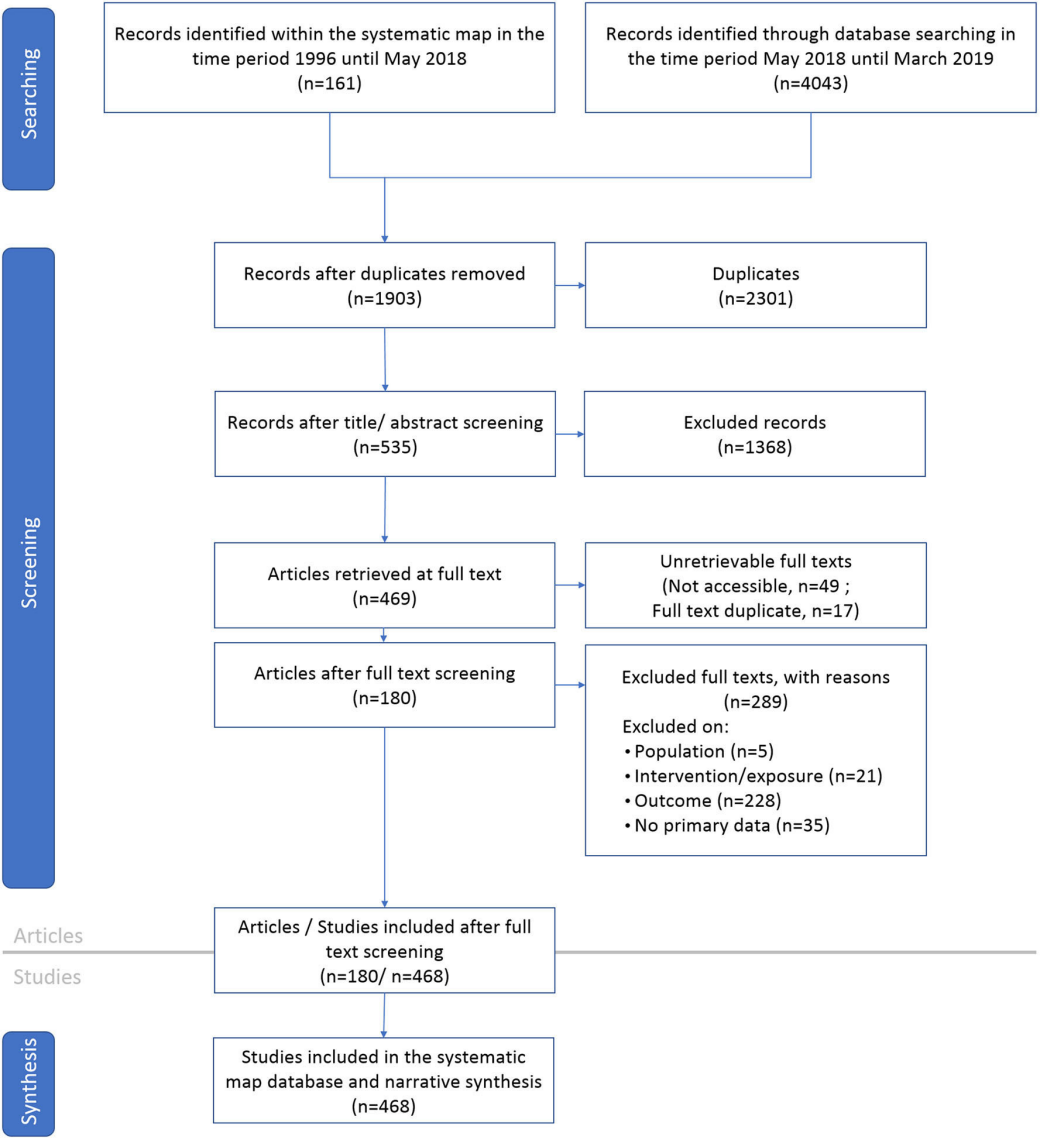
Flow diagram of the systematic mapping process explaining the selection of relevant articles and studies. This diagram follows ROSES guidance (Haddaway et al., 2018).

The CRISPR/Cas articles analyzed to answer secondary question 2 of the systematic map about the occurrence of off-target effects were included in this systematic review, covering the time period between 1996 and May 2018 (a list is provided in additional file 6 of the published systematic map; Modrzejewski et al., 2019). In addition, the literature search was updated in March 2019, to identify all CRISPR/Cas articles published between May 2018 and March 2019 (Figure 1). The search string was similar to the search string of the systematic map but focused on CRISPR/Cas (the question addressed in the systematic map was broader in scope and included besides to CRISPR/Cas also other genome editing techniques). It comprises two parts: The first part defined the population of interest comprising less specific terms like crop, plant or seed as well as specific model plants and crops including their English and Latin names. The second part defined the intervention, comprising different CRISPR/Cas variants. The search terms within each class were combined using the Boolean operator “OR”; the two classes were combined using the Boolean operator “AND.” The final search string for Web of Science is provided in Supplementary Table 1. The search string was adapted to the specific needs of each database.

The following online publication databases and platforms were searched for academic literature. Access was ensured by institutional subscriptions:

- Web of Science (WoS), including Web of Science Core Collection, BIOSIS Citation Index, BIOSIS Preview, CABI:CAB Abstracts and Global Health, Data Citation Index, Derwent Innovations Index, KCI-Korean Journal Database, Russian Science Citation Index, SciELO Citation index, Zoological Records
- Scopus
- PubMed
- Science direct
- AGRIS

Furthermore, Google Scholar (https://scholar.google.com/) was searched using 30 different combinations of the most relevant (model) plants and CRISPR/Cas terms. The first 20 search results, organized by relevance, of each combined search string were assessed at the title/abstract level. Deviating from the systematic map protocol, no search for gray literature on company webpages was conducted, because we noticed that they do not provided any data about the occurrence of off-target effects (Modrzejewski et al., 2019). All hits from each database were imported into an EndNote X8.0.1 library file. Duplicates were removed using the appropriate function within the EndNote software. Afterwards, the remaining records were imported into the open-access and non-profit database CADIMA to increase transparency and traceability during the review process (Kohl et al., 2018).

### Article Screening and Study Inclusion Criteria

#### Screening Process

When conducting the systematic map a consistency check was performed aiming to assure for a good inter-reviewer agreement. As the review team stayed the same and the eligibility criteria were nearly identical compared to the systematic map (except that only CRISPR/Cas articles were an eligible intervention and not of all genome editing techniques) we did not carry out a further consistency check/kappa test within this review. Two reviewers conducted title/abstract screening independently. Two reviewers then checked the potentially relevant articles at full text level. Lists of unobtainable articles and articles excluded at full text level with the reason for exclusion are provided (Supplementary Table 2). All members of the review team are authors of a few articles retrieved by the literature search. However, as none of their articles provides primary data, these articles were excluded at title/abstract level.

#### Eligibility Criteria

In order to be included in the systematic review each article had to meet the following inclusion criteria:

*Eligible population*: Any model plant or crop produced for agricultural production.

*Eligible intervention*: The CRISPR/Cas technique was used to induce any on-target mutation.

*Eligible outcome*: The occurrence of off-target effects was assessed using either biased or unbiased detection methods. In case no potential off-target sequences were identified using prediction tools and thus no potential off-target sequences were tested for the occurrence of off-target effects, the study was excluded.

*Eligible type of data*: Only those references were included which comprise primary data referring to the use of a CRISPR/Cas and the detection of potential off-target effects.

*Eligible languages*: References in German and English language were included. Articles in other languages were included when besides title and abstract, further parts of the article, like figures or tables, were in English or German and the provided information allowed for a definite judgement of their relevance.

#### Data Coding Strategy

One article can contain several studies e.g., when different plant species were investigated or different sgRNA, nucleases or delivery methods were applied. While articles were screened for relevance at title/abstract and full text level, relevant data were finally extracted at study level. Due to limited available resources, one reviewer extracted the relevant data. Unclear cases were marked by the first reviewer and cross-checked by another one. Building up on the data extracted within the systematic map, further details were extracted for the systematic review as shown in Table 1. The following data of each included study were extracted into one row in an excel sheet (each in a cell):

**Table 1:** Overview about the extracted data within the systematic map and further data that were additionally extracted for the systematic review.

	**Data extracted within the systematic map**	**Further data additionally extracted for the systematic review**
Bibliographic information	- Abstract - Authors - DOI/ISBN - Issue number - Keywords - Page range - Periodical - Title - Volume - Year	None
Population	Plant species	None
Intervention	Genome editing technique (e.g., CRISPR/Cas)	- Specification of the nuclease variant (e.g., SpCas9, SaCas9, Cpf1)
Transformation	None	- Delivery method (DNA-stable, DNA-transient, RNA, Ribonucleoproteins) (RNP)
Protospacer	None	Guanin-cytosine (GC)-content of the target sequence
Target sequence	Sequence identifier (Name of the gene)	- Different sgRNAs targeting one gene were extracted separately (each sgRNA in one row)
Off-target prediction	- Number of identified potential off-target sequences - Prediction tool	None
Off-target detection	- Detection method	None
	- Number of analyzed off-target sequences	- Number of mismatches - Position of mismatches in relation to the PAM
Identified off-target effects	Off-targets identified (Yes/No)	- Number of identified off-targets, - Number of mismatches - Position of mismatches in relation to the PAM

In case relevant data were missing or not accessible, the corresponding author of the article was asked to provide the missing information within a period of 2 weeks. The extracted data are available in Supplementary Table 3 (summary) and Supplementary Table 4 (details of all potential off-target sequences).

#### Study Validity Assessment

Articles that passed the study inclusion criteria were subjected to critical appraisal. The critical appraisal was conducted on a study-by-study basis. Criteria were defined within the review team in order to assess internal validity (risk of bias within a specific study) and external validity (generalizability). These criteria reflect critical variables that affect the reliability of study outcomes. The critical appraisal criteria are listed in Table 2. Each study was assessed by considering its compliance with each of the developed criteria (yes, partly, no, unclear). Based on these judgments, studies were categorized as having *high, medium, low* or *unclear validity*. In case that, due to the fact that more than one potential off-target sequences with diverging quality were included in one study, a study was considered as being of *medium validity* in a first step. In a next step the medium category was further subdivided into medium/high or medium/low depending on the characteristics of the individual sequence (for more detail also see Table 3). This may occur in case within one study (i) several potential off-target sequences were analyzed but only some of them were followed by a PAM, (ii) information were solely provided for a subset of analyzed potential off-target sequences or (iii) a subset of analyzed potential off-target sequences were identical to the on-target site. All potential off-target sequences within one *medium-study* that fulfilled the required criteria were moved to the *medium/high* category, while the remaining ones were rated as *medium/low validity* according to Tables 2, 3. All studies were included in the narrative report, but based on the overall ranking of the critical appraisal, the *high* and *medium/high validity* categories were handled separately from the *low* and *medium/low* as well as the *unclear validity* studies. Due to limited available resources, solely one reviewer assessed the validity of all studies. Doubtful cases were discussed within the whole review team. The results of the critical appraisal are detailed in Supplementary Table 5.

**Table 2:** Study validity assessment criteria.

**Question/criterion**	**Yes**	**Partly**	**No**	**Unclear**
**Study design/methods used**				
Did the CRISPR/Cas technique induce any on-target mutation?	Any on-target mutation was induced	–	No on-target mutation was induced	Lacking sufficient information to judge
Is a reference genome of the edited plant available?	Reference genome is available	–	Reference genome has not been fully sequenced/Sequencing error when analyzing potential off-target sequences	Lacking sufficient information to judge
**Potential off-target sequences**				
Did a PAM follow the potential off-target sequences?*	All potential off-target sequences were followed by a PAM	Some potential off-target sequences were followed by a PAM	No potential off-target sequences were followed by a PAM	Lacking sufficient information to judge
Has the potential off-target sequence at least one mismatch to the on-target site	All potential off-target sequences have at least one mismatch	Some potential off-target sequences have at least one mismatch	All potential off-target sequences have no mismatch	Lacking sufficient information to judge
Are information about the potential off-target sequences provided? (number of mismatches, the position of mismatches to the PAM)	All information are provided	Information are partly provided	No information are provided	–

**Table 3:** Overall assessment of study validity.

**Studies were assigned *low validity* or *unclear validity* if any of the following factors applied**
**Any of these criteria answered with “No” or “Unclear”**
Did the CRISPR/Cas technique induce any on-target mutation?
Did a PAM follow the potential off-target sequences?
Has the potential off-target sequence at least one mismatch to the on-target site?
**Any of these criteria answered with “No”**
Is a reference genome of the edited plant available?
Did sequencing errors occur?
Are information about the potential off-target sequences provided? (number of mismatches, the position of mismatches to the PAM)
**Studies that were not assigned** ***low validity*** **were considered to have** ***medium validity*** **if any of the following factors applied**
**Any of the criteria answered with “Partly”**
Did a PAM follow the potential off-target sequences?
Has the potential off-target sequence at least one mismatch to the on-target site
Are information about the potential off-target sequences provided? (number of mismatches, the position of mismatches to the PAM)
**Potential off-target sequences within** ***medium validity*** **studies were handled as** ***medium/low validity*** **if any of the following factors was not applied**
**Any of the criteria answered with “No”**
Did a PAM follow the potential off-target sequences?
Has the potential off-target sequence at least one mismatch to the on-target site
Are information about the potential off-target sequences provided? (number of mismatches, the position of mismatches to the PAM)
**Potential off-target sequences within** ***medium validity*** **studies were handled as** ***medium/high validity*** **if all the following factors applied**
**All of the criteria answered with “Yes”**
Did a PAM follow the potential off-target sequences?
Has the potential off-target sequence at least one mismatch to the on-target site
Are information about the potential off-target sequences provided? (number of mismatches, the position of mismatches to the PAM)

One reviewer answered the questions with “Yes,” “Partly,” “No,” or “unclear.” The questions were answered based on the specification in the table. Questions answered with “Yes” means that the study has a *high validity* (low risk of bias) regarding this criteria, while “No” means that the study has a *low validity* (high risk of bias). Questions answered with “Partly” means that a subset of analyzed potential off-target sequences within this study has a *high validity* and a subset has a *low validity* regarding this criteria. ^*^ CRISPR/SpCas9 and engineered SpCas9 nuclease variants: NGG-PAM or NAG-PAM or NGA-PAM (VQR-variants): CRISPR/Cpf1: TTN-PAM or TTTN-PAM; CRISPR/SaCas9 and CRISPR/eSaCas9: NNGRRT-PAM.

If none of the above categories applied, the study was considered as *high validity*.

#### Potential Effect Modifiers/Reasons for Heterogeneity

The following potential effect modifiers were considered for studies included in this systematic review:

- *Number of mismatches*: It is estimated that the number of mismatches between the target sequence and the potential off-target sequence significantly affects the occurrence of off-target effects.
- *Position of mismatches proximal to the PAM*: Mismatches occurring within the seed sequence (8 up to 12 nucleotides proximal to the PAM) may decrease off-target effects compared to when the mismatch is located more distant to the PAM.
- *GC-content of the targeting sequence*: The GC-content of a chosen target sequence may affect off-target effects, as high GC-content stabilize the sgRNA/genomic DNA hybridization (Fu et al., 2013). Therefore, a low GC-content may result in decreased off-target activity.
- *Altered nuclease variants*: Next to the most widely used nuclease Cas9 derived from *Streptococcus pyogenes* (SpCas9) several further Cas9 proteins from different bacterial or archaea species have been adapted (e.g., SaCas9, SpCas9-HF, Cpf1, Nickase).
- *Time of incubation*: Time during which genomic DNA is exposed to CRISPR/Cas (Stable or transient transformation with respective DNA sequences, RNA sequences or RNP).

Further potential effect modifiers were identified but not systematically assessed within this review:

- Different plant species (due to genome size and ploidy level)
- Prediction tools used to identify predicted potential off-target sequences
- Detection method used to identify off-target effects

#### Data Synthesis and Presentation

All studies included in the systematic review were synthesized in a narrative report. No studies were excluded, but studies rated as *high* and *medium/high validity* were reported separately from studies rated as *low* and *medium/low* or *unclear validity*.

All analyzed potential off-target sequences identified within the high and the *medium/high validity* studies were included to address five different hypothesis:

*Hypothesis 1*: *An increasing number of mismatches between the on-target site and the potential off-target sequence decreases the likelihood that off-target effects occur*.

*Hypothesis 2*: *Mismatches within the first eight nucleotides proximal to the PAM sequence (seed sequence) decrease the occurrence of off-target effects*.

*Hypothesis 3: A lower guanine-cytosine (GC)-content of the target sequence decrease the occurrence of off-target effects*.

*Hypothesis 4*: *Improved nuclease variants decrease the occurrence of off-target effects*.

*Hypothesis 5*: *The period in which the CRISPR/Cas system is active in the plant cell affects the occurrence of off-target effects*.

For all hypotheses, a descriptive overview of the available literature was given considering all *high* and *medium/high validity* studies. In addition, a quantitative synthesis was performed. Results of studies rated as *low* or *medium/low validity* as well as *unclear validity* studies were narratively reported but no specific validation regarding the formulated hypotheses was performed. Instead, based on the reasons for study validation as low and medium/low or unclear it was described whether off-target effects were detected or not.

#### Quantitative Synthesis Strategy

Three hundred and sixty three out of 370 studies rated as high or medium/high validity were used for quantitative synthesis. Seven studies analyzing 5,021 potential off-target sequences were not included for quantitative synthesis as unbiased detection methods were used to detect off-target effects. Due to the huge amount of analyzed potential sequences it was not possible to extract all mismatches including their position proximal to the PAM in detail. In none of these sequences, off-target effects were detected. Four potential off-target sequences contained three mismatches, while the remaining ones had at least four mismatches to the on-target sequence. Detailed information about the sequences included in this meta-analysis as well as a list of excluded sequences is provided in Supplementary Table 6. We ran a binary logistic regression analysis to verify the five hypothesis to answer the question, which factors affect the occurrence of off-target effects by using CRISPR/Cas in plants. This type of analyses aims to investigate the relationship between a set of independent variables and the depended response variable and provides information about the probability of the response of interest, the independent variables which mostly affect the response of interest and the odd ratios (Walsh, 2016). As indicated in Table 4, number of mismatches (x1 = 1/2/3/≥4), position of mismatch/es proximal to the PAM (x2 = Yes/No), GC-content [x3 = high (>50%)/low (<50%)], delivery method (x4 = DNA-stable/others) and nuclease variant (x5 = SpCas9/further nucleases) were considered as independent variables (x = classification). Moreover, the occurrence of an off-target effect (Y = Yes/No) were applied as depended variable. The Fit Model platform implemented in the software package JMP (JMP®, 2019. *JMP®. Version 14. SAS Institute Inc. Cary, NC*.) was used to fit a binary logistic regression model by using the binary logistic personality(SAS Institute, 2018. *JMP® 14 Fitting Linear Models*). Therefore, the Yes level of the dependent variable was defined as response level i.e., the model estimates the probability of the Yes level of the off-target-effect variable (SAS Institute, 2018. *JMP® 14 Fitting Linear Models*). First, a model was fitted including all independent variables described in Table 4 (“full model”). All variables that appear insignificant based on effect likelihood ratio chi-square test were excluded. Then, a second model (“reduced model”) was fitted which includes all significant variables of the full model. Again, all variables that appear insignificant were excluded resulting in a final model (“final model”) obtaining the significant variables. The Fit Model platform provides three tests to evaluate the model fit: (i) whole model test, (ii) lack of fit test, and (iii) likelihood ratio effect test (SAS Institute, 2018. *JMP® 14 Fitting Linear Models*). Additionally, to evaluate the accuracy and predictive ability of the final model, a confusion matrix was created (SAS Institute, 2018. *JMP® 14 Fitting Linear Models*) and sensitivity (true positive rate) and specificity (false positive rate) were calculated (Walsh, 2016). Furthermore, a receiver operating characteristic (ROC) curve was created and the area under the curve (AUC) was calculated. A ROC curve greatly exceeds the diagonal and a high AUC value indicates a model with good accuracy and a high predictive ability (Walsh, 2016). Furthermore, odds ratios were estimated based on the final model.

**Table 4:** Description of independent Variables.

**#**	**Variable**	**Variable description**	**Level**	**Number of analyzed potential off-target sequences**	**Total (%)**
x1	Number of mismatches	One mismatch	1	154	11
		Two mismatches	2	218	16
		Three mismatches	3	352	25
		Four or more mismatches	≥4	671	48
x2	Position mismatch/es proximal to the PAM	(Any) mismatch within the seed sequence (Position 1-8 proximal to the PAM)	Yes	862	62
		Any mismatch/es within the seed sequence	No	533	38
x3	GC-content	<50%	Low	505	36
		≥50%	High	890	64
x4	Delivery method	DNA-stable	DNA-stable	1,229	88
		Others (DNA-transient, RNP, RNA)	Others	166	12
x5	Nuclease variant	CRISPR/SpCas9	SpCas9	1,218	87
		Further Nucleases	Others	177	13

## Results

### Review Descriptive Statistics

#### The Evidence Base

Our systematic review included in total 468 studies from 180 articles. Figure 1 presents a flow diagram of the systematic review process with the number of articles and studies included and excluded at each stage. One hundred and thirty three articles (306 studies) were derived from the systematic map (secondary question 2) that preceded this review (Modrzejewski et al., 2019). Twenty eight articles from the systematic map were not rated eligible for inclusion in this review because of ineligible intervention (*n* = 19) or ineligible outcome (*n* = 9). The literature update identified further 47 articles (162 studies) for the period May 2018 until March 2019. A list of all articles and studies comprised in this systematic review is provided in Supplementary Table 3 and includes the extracted data.

#### Characteristics of Articles Included in Narrative Synthesis

All articles included in this systematic review were published in peer-reviewed journals in English language.

#### Articles per Year

In 2013, the first four articles were identified that assessed off-target effects in plants after applying CRISPR/Cas. Since that time, the number of such articles on CRISPR/Cas increased continuously to 62 in 2018. In the first quarter in 2019, the number of relevant articles reached 21.

#### Study Design

We identified five different possibilities how potential off-target sequences have been identified and analyzed:

- Potential off-target sequences that are similar to the target sequence were identified using bioinformatics approaches (mainly CRISPR-P, *n* = 104; BLAST, *n* = 61, and Cas-OFFinder, *n* = 55). Then a subset of potential off-target sequences with the considered highest likelihood for occurrence of off-target effects were analyzed using biased detection tools (mainly PCR + Sequencing).
- Potential off-target sequences that are similar to the target sequence were identified using bioinformatics approaches. All potential off-targets sites with up to a certain number of mismatches were analyzed for the occurrence of off-target effects.
- Potential off-target sequences for the chosen target sequence were already known (for example due to polyploidy of plants or due to a priori research). Only these sequences were analyzed for the occurrence of off-target effects.
- To analyze the binding specificity of the CRISPR/Cas system, variations were made in the protospacer binding site. The target site was then assessed for the occurrence of mutations.
- The whole genome was sequenced for the occurrence of off-target effects.

#### Results Critical Appraisal

Three hundred and twenty six out of 468 studies were rated *high validity*, 44 to have *medium/high* and *medium/low validity*, 72 to have *low validity*, and 26 to have *unclear validity*. One study potentially rated as *high validity* was downgraded to *low validity* because the sgRNA/Cas9 complex was constitutively overexpressed but it is not traceable how this overexpression was actually achieved (Ji et al., 2018). Detailed information about the validity assessment of all studies including the reason for their classification is provided in Table 5 and Supplementary Table 5. Within the studies rated as *high validity*, 2,267 potential off-target sequences were assessed for the occurrence of off-target effects. In addition, 4,149 potential off-target sequences identified from 44 studies rated as *medium validity* fulfilled the required criteria and were rated as *medium/high validity* [(i) sequences were followed by a PAM (ii) All information were given (iii) sequences had at least one mismatch to the on-target sequence]. Therefore, the database of *high* and *medium/high validity* studies comprises 370 on-target sequences and in total 6,416 potential off-target sequences were analyzed to identify off-target effects. Within the studies rated as *low validity*, 197 potential off-target sequences were assessed for the occurrence of off-target effects. In addition, 154 potential off-target sequences identified from 44 studies were rated as *medium/low validity* as they didn't fulfill the required criteria [(i) sequences were not followed by a PAM (ii) Information are incomplete (iii) on-target and off-target site were identical]. Adding these sites in total, 351 potential off-target sequences were rated as *low* or *medium/low validity*. One hundred and seventy potential off-target sequences lack sufficient information and were rated as *unclear validity*.

**Table 5:** Overview on the validity assessment of all studies and the reason for validation.

**Validity assessment**	**Number of studies**	**Reason for validity assessment outcomes**	**Number of analyzed potential off-target sites**
*High validity*	326	All study validity assessment criteria were rated as “Yes”	*High validity*: 2,267
*Medium validity*	44	Some potential off-target sequences were followed by a PAM (*n* = 28) Information are considerably incomplete (*n* = 15) Only some potential off-target sequences show at least one mismatch (*n* = 7)	*Medium/High validity*: 4,149 *Medium/Low validity*: 154
*Low validity*	72	No on-target mutation was induced (*n* = 27) Reference genome has not been fully sequenced/sequencing error when analyzing potential off-target sequences (*n* = 7) Potential off-target sequences were not followed by a PAM (*n* = 15) All potential off-target sequences have no mismatch (*n* = 5) No information is provided (*n* = 23) Overexpression of the sgRNA/Cas9 complex (*n* = 1)	*Low validity*: 197
*Unclear validity*	26	Lacking sufficient information to judge (*n* = 26)	*Unclear validity*: 170

#### Characteristics of High and Medium/High Validity Studies

In total, 370 studies were rated as having *high* or *medium/high validity*. These studies analyzed a total number of 6,416 potential off-target sequences for the occurrence of off-target effects. Table 6 summarizes key characteristics of these analyzed sequences, including the applied nuclease variant, delivery method and the number of analyzed potential off-target sequences. Thirteen different nuclease variants were identified that were applied and assessed for off-target effects in plants. Mostly the nuclease SpCas9 was used (97%) followed by Cpf1 (1%), eSpCas9 (1.0) (0.5%), eSpCas9 (1.1) (0.5%), and SaCas9 (0.5%).

**Table 6:** Overview about the characteristic items and frequency of studies rated as *high* and *medium/high validity*.

**Study validity assessment**	***n*=**	**Nuclease variant**	***n*=**	**Delivery method**	***n*=**	**Analyzed potential off-target sequences**
*High validity* and	370	SpCas9	311	DNA-stable	283	5,999
*medium/high validity*						
				DNA-transient	18	78
				RNPs	8	133
				RNA	2	9
		Cpf1	20	DNA-stable	20	62
		eSpCas9 (1.1)	8	DNA-stable	4	26
				DNA-transient	4	7
		eSpCas9 (1.0)	8	DNA-stable	3	24
				DNA-transient	5	8
		SaCas9	6	DNA-stable	2	5
				DNA-transient	4	4
		Others	17	DNA-stable	9	47
				DNA-transient	8	14

*RNP, Ribonucleoproteins; RNA, Ribonucleic acid*.

In the large majority, the CRISPR/Cas system was delivered into the cells via stable transformation of DNA (321 studies). Thirty nine studies transiently supplied the CRISPR/Cas system as plasmid DNA (*DNA-transient*). Eight studies used a DNA-free approach by suppling the CRISPR/Cas system as ribonucleoproteins (*RNP*) and two studies delivered the CRISPR/Cas system via RNA sequence. More than 93% of the analyzed potential off-target sequences were assessed when using the standard nuclease SpCas9 and the system was supplied into the cell as stably integrated DNA approach (*DNA-stable*).

Based on the results gained within the *high* and *medium/high validity* studies, we conducted a meta-analysis and assessed five different hypothesis about the occurrence of off-target effects in CRISPR/Cas studies in plants. In addition, a descriptive overview based on the identified evidence base is provided for each hypothesis.

### Meta-Analysis of *High* and *Medium/High Validity* Studies

**Figure 2:**
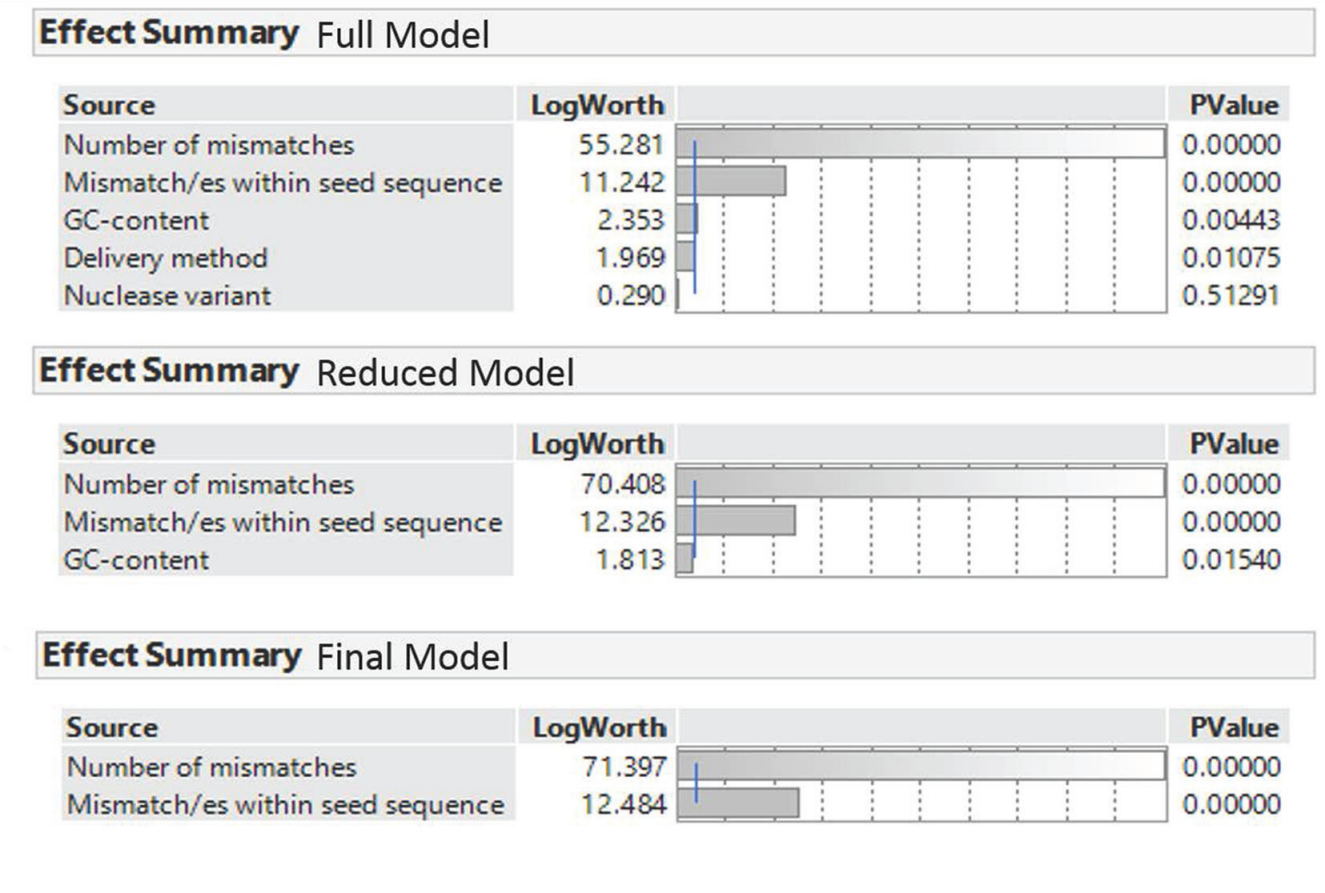
Effect summary of the binary regression analysis for the full model, the reduced model, and the final model. The LogWorth transformation adjusts *P*-value to provide an appropriate scale for graphing. A value that exceeds 2 is significant.

Binary regression analysis was conducted to evaluate if the independent variables “number of mismatches,” “position of mismatches proximal to the PAM,” “GC-content,” “nuclease variant,” and “delivery method” affect the occurrence of off-target effects. In a first step, a full model was fitted including all variables as indicated in Table 4. Then, the full model was reduced by removing the nuclease variant and the delivery methods, as these variables appeared to be insignificant (reduced model) (Figure 2). In a next step, a reduced model was fitted including the remaining variables. Due to insignificance, the reduced model was further reduced by removing the variable GC-content as it appeared to be insignificant. Therewith we gained the final model including the variables number of mismatches and position of mismatches proximal to the PAM (Figure 2). The final binary regression analysis revealed that the number of mismatches and the position of mismatches proximal to the PAM significantly affect the occurrence of off-target effects, whereby the number of mismatches has the dominant impact (Figure 2).

The whole model test, lack of fit test and likelihood ratio effect test indicate that the final model fitted the data in an appropriate and sufficient way (details are provided in Supplementary Table 6). Furthermore, the observed accuracy rate indicated that the final model predicted 92% of the recorded off-target effects correctly i.e., correct assignment to the “Yes” or “No” level of the dependent variable. In detail, the amount of true negatives was higher than the amount of true positives, indicated by a specificity of 0.98 and a sensitivity of 0.46. Due to the bias in specificity and sensitivity, which can be caused by unbalanced ratio between the Yes and No level of the dependent variable, a ROC curve was additionally used to evaluate the model fit. The ROC curve exceeded the diagonal and the corresponding AUC value (0.92) indicates a model with good accuracy and a high predictive ability (Supplementary Table 6). To verify the five hypotheses odds ratios were calculated for the variables with the final model.

Hypothesis 1: *An increasing number of mismatches between the on-target and the potential off-target sequence deceases the probability that off-target effects occur*.

The probability of the occurrence of an off-target effect if the number of mismatches is one is 3.8, 36.7, or 160.6 times higher than if the number of mismatches is 2, 3, or ≥4, respectively (Table 7A). Therefore, the probability of the occurrence of an off-target effect is highest for number of mismatches = 1 and is lowest for the number of mismatches = ≥4. To conclude based on the odds ratios calculated for the final model hypothesis 1 can be confirmed.

**Table 7:** Odds ratios for number of mismatches and mismatch/es within seed sequence.

**Level 1**	**Level 2**	**Odds Ratio**	**Prob>Chisq**	**Lower 95%**	**Upper 95%**
**A) Number of mismatches**					
2	1	0.2661273	<0.0001	0.1661508	0.4262619
3	1	0.0272239	<0.0001	0.0135418	0.05473
3	2	0.1022967	<0.0001	0.0515393	0.2030414
≥4	1	0.0062283	<0.0001	0.0024145	0.016066
≥4	2	0.0234035	<0.0001	0.009159	0.0598016
≥4	3	0.2287805	0.0069	0.0784442	0.6672328
1	2	3.7576002	<0.0001	2.3459754	6.0186306
1	3	36.732378	<0.0001	18.271515	73.845412
2	3	9.7754885	<0.0001	4.9251049	19.402668
1	≥4	160.55728	<0.0001	62.243085	414.16069
2	≥4	42.728674	<0.0001	16.72197	109.18209
3	≥4	4.3710014	0.0069	1.4987273	12.747919
**B) Mismatch/es within seed sequence**					
No	Yes	0.48958322	<0.0001	3.1263158	7.6669071
Yes	No	0.2042554	<0.0001	0.1304307	0.3198653

Hypothesis 2: *Mismatches within the first eight nucleotides proximal to the PAM decrease the occurrence of the off-target effect*.

The probability of the occurrence of an off-target effect if the mismatches are not within the first eight nucleotides proximal to the PAM is 4.9 times higher than if the mismatches are within the first eight nucleotides proximal to the PAM (Table 7B). Therefore, based on the odds ratios calculated for the final model hypothesis 2 can be confirmed.

Hypothesis 3: *A lower GC-content of the target sequence decrease the occurrence of off-target effects*.

The variable GC-content did not significantly contribute to the fitted model and were not included as independent variable in the binary logistic regression analysis. Therefore, it is not possible to confirm this hypothesis.

Hypothesis 4: *Improved nuclease variants decrease the occurrence of off-target effects*.

The variable nuclease variant did not significantly contribute to the fitted model and were not included as independent variable in the binary logistic regression analysis. Therefore, it is not possible to confirm this hypothesis.

Hypothesis 5: *The period in which the CRISP/Cas construct is active in the plant cell affects the occurrence of off-target effects*.

The delivery method did not significantly contribute to the fitted model and were not included as dependent variable in the binary logistic regression analysis. Therefore, it is not possible to confirm this hypothesis.

### Descriptive Synthesis of *High* and *Medium/High Validity* Studies

The 370 studies rated as *high* or *medium/high validity* assessed a total amount of 6,416 potential off-target sequences. One hundred and fifty four analyzed potential off-target sequences showed one mismatch (2.4% of the total analyzed sequences), 218 had two mismatches (3.4%), 356 had three mismatches (5,6%), while the remaining 5,688 sequences (88,7%) referred to four or more mismatches.

### Hypothesis 1: Number of Mismatches

**Hypothesis 1:** *An increasing number of mismatches between the on-target site and the potential off-target sequence decreases the likelihood that off-target effects occur*.

It is purported that the main factor to control off-target effects is a careful selection of the target sequence (Hsu et al., 2013; Zhu et al., 2017; Zischewski et al., 2017). This hypothesis aims to assess the occurrence of off-target effects depending on the number of mismatches between the on-target and potential off-target sequences.

**Figure 3:**
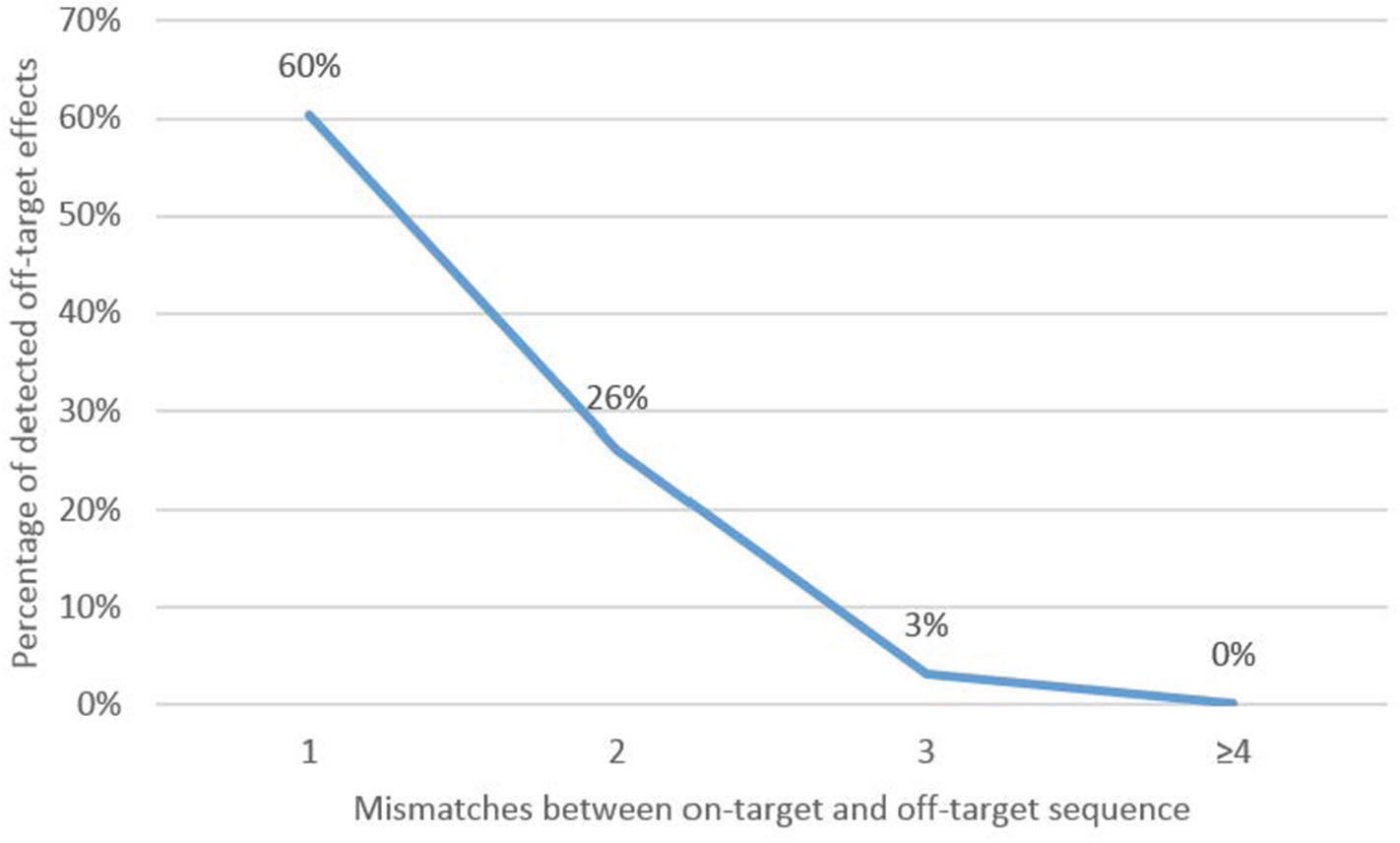
Percentage of detected off-target effects for one (*n* = 154), two (*n* = 218), three (*n* = 356), and four or more (*n* = 5,688) mismatches. Calculation: Number of detected off-target effects/number of analyzed potential off-target sequences.

As shown in Figure 3, the number of identified off-target effects steeply decreases the more mismatches occur between the on-target and potential off-target sequences. One mismatch in respect to the on-target sequence lead to the detection of off-target effects in 93 out of 154 analyzed potential off-target sequences (59%). Two mismatches decreased the occurrence of off-target effects to 26% (57 identified off-target effects out of 218 analyzed potential off-target sequences). Three mismatches between the on-target and potential off-target sequence decreased the off-target rate to 3% (11 identified off-target sequences out of 356 analyzed potential off-target sequences). Four or more mismatches further decreased the off-target rate to 0.09% (five off-target effects out of 5,688 analyzed potential off-target sequences). The five identified off-target effects had two times four and five mismatches each and once six mismatches.

#### Conclusions for Hypothesis 1

The data clearly indicate that the likelihood of off-target effects can be minimized drastically by selecting a target sequence that shows more than three mismatches to any similar sequences in the genome.

#### Consequences of Hypothesis 1 for the Evaluation of the Hypotheses 2–5

The results for the first hypothesis show that the number of mismatches to the target sequence has a very strong influence on the occurrence of off-target effects. In addition, it was shown that off-target effects occur at considerable low likelihood when the potential off-target sequences bear at least four mismatches. Therefore, the hypotheses 2–5 will be examined separately for one and two mismatches to elucidate a consideration independently from the interfering number of mismatches. In addition, detected off-target effects containing three or more mismatches to the target sequence will be reviewed though the likelihood of an effect is generally low.

### Hypothesis 2: Position of Mismatches

**Hypothesis 2**: *Mismatches within the first eight nucleotides proximal to the PAM sequence decrease the occurrence of off-target effects*.

It was considered that mismatches occurring within the first nucleotides proximal to the PAM determine the occurrence of off-target effects, leading to a decreased or even complete abolition of off-target effects (Hsu et al., 2013; Endo et al., 2015; Hahn and Nekrasov, 2018). This hypothesis aims to assess the occurrence of off-target effects considering the position of the mismatch/es in potential off-target sequences.

#### One Mismatch Between the On-Target and the Potential Off-Target Sequence

**Figure 4:**
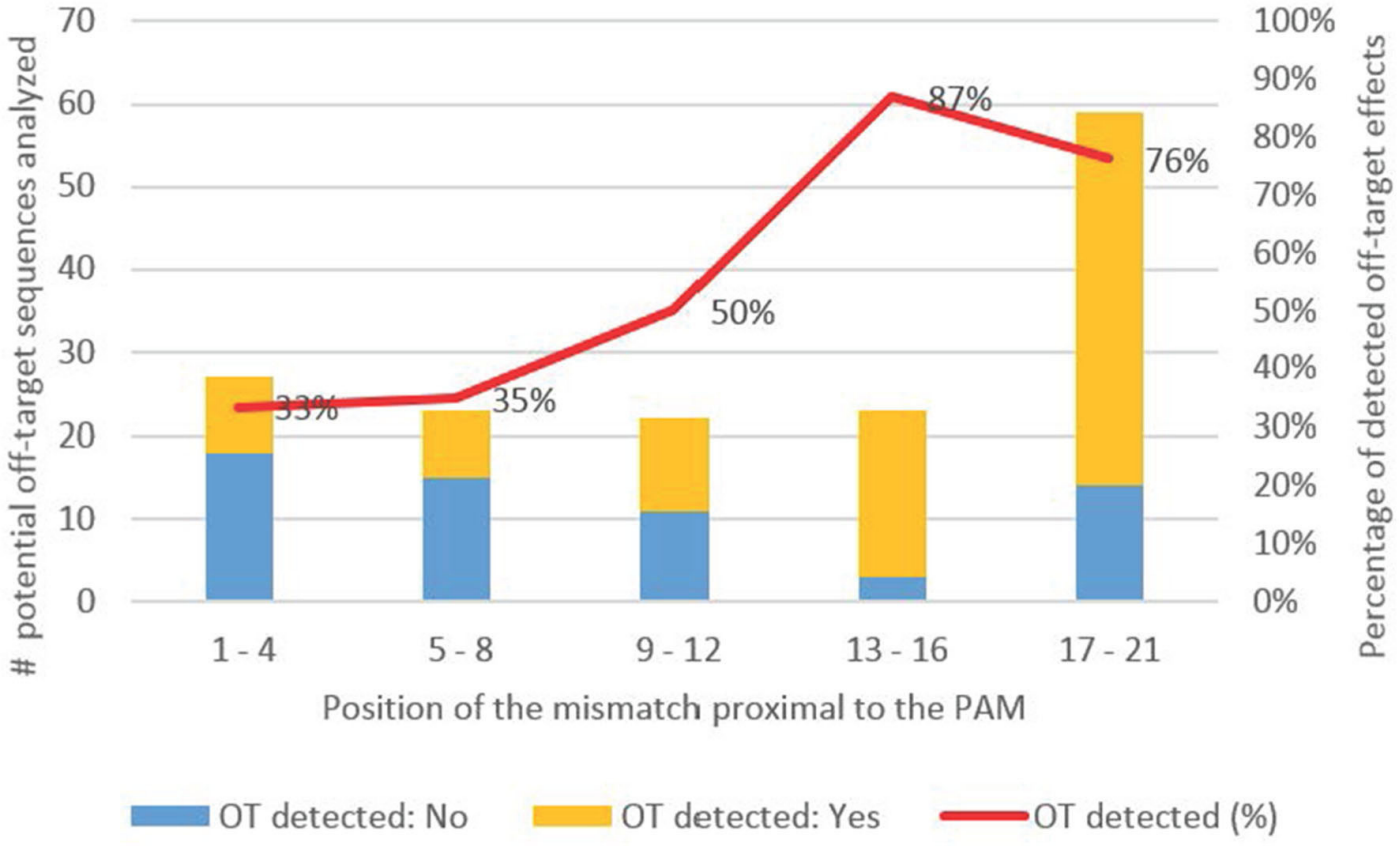
Percentage of detected off-target effects for one mismatch based on the position the mismatch is located proximal to the PAM. The guide sequence is divided in five intervals of four nucleotides each (Position 1–4 proximal to the PAM, position 5–8, 9–12, 13–16, 17–21. Yellow: Number of studies with detected off-target effects; blue: Number of studies without detected off-target effects; Red: Percentage of detected off-target effects for each interval.

In total, 154 potential off-target sequences with one mismatch were assessed for off-target effects. Because of the limited set of data, we analyzed the incidences of off-target effects based on the position of the mismatch proximal to the PAM in five intervals of four nucleotides each covering the whole guide sequence (Figure 4). If the mismatch occurred at position one to four proximal to the PAM, off-target effects were detected in 33% of the analyzed sites [nine off-target effects identified out of 27 potential off-target sequences (9/27)]. A mismatch at position five to eight proximal to the PAM showed a similar off-target rate of 35% (8/23). The off-target rate considerably increased to 50% when the mismatch was located at position 9–12 (11/22) and up to 87% in case the mismatch was located at position 13–16 (20/23). A high off-target rate of 76% (45/59) was also observed when the mismatch was located at position 17–21 to the PAM.

#### Two Mismatches Between the On-Target and the Potential Off-Target Sequences

**Figure 5:**
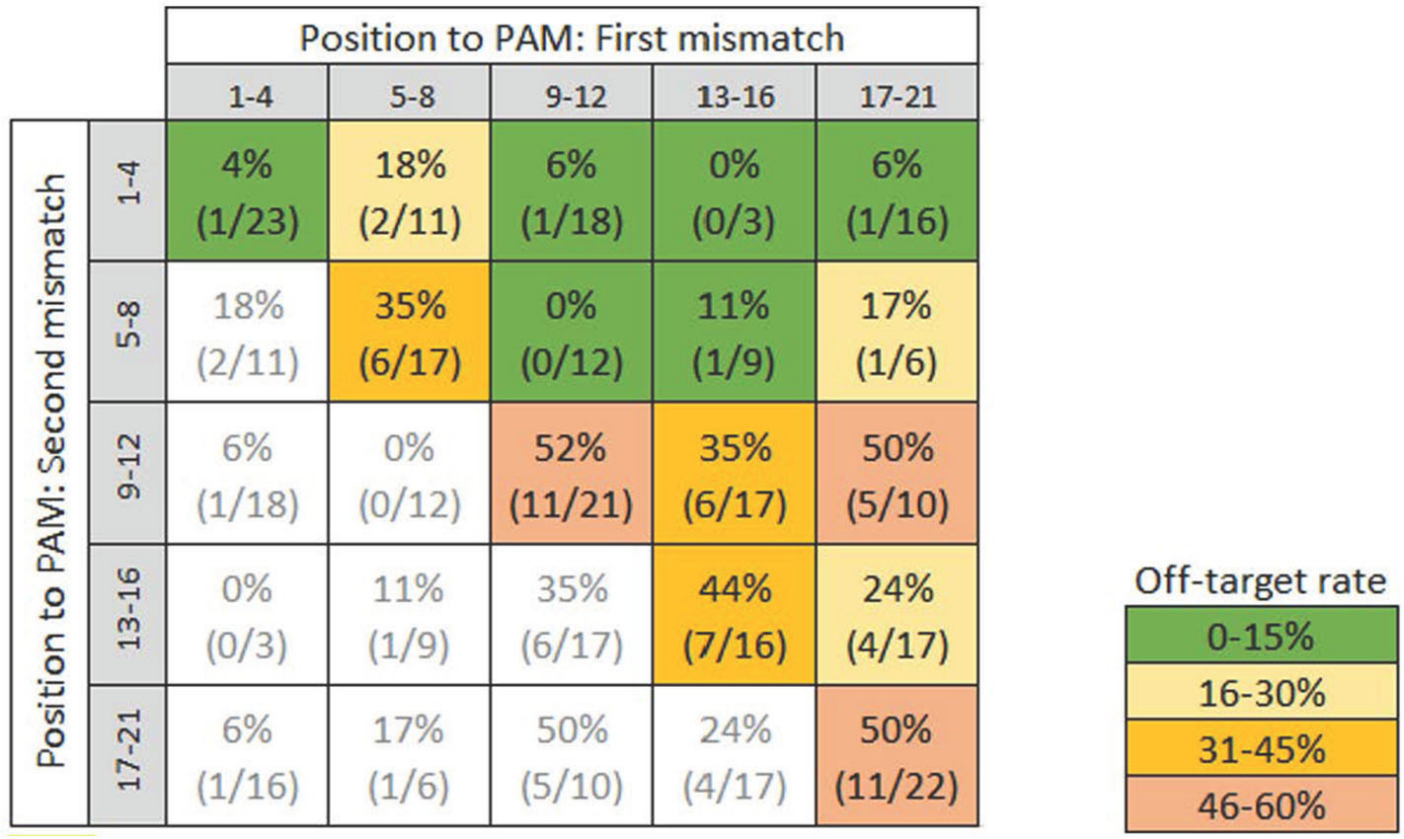
Incidence of off-target effects when two mismatches occur proximal to the PAM. (x/y): (“x” off-target effects identified/“y” potential off-target sequences analyzed).

Two hundred and eighteen potential off-target sequences with two mismatches were assessed for off-target effects. We draw a cross table to display the off-target rate based on the position of the two mismatches proximal to the PAM within the intervals as before (Figure 5). The results indicate that the further the mismatches are distant from the PAM the higher the off-target rate. The results also indicate that the off-target rate increases when the two mismatches are located close to each other. When both mismatches are located within the seed sequence at positions five to eight, off-target effects occurred in 35% of the analyzed potential off-target sequences (six off-target effects were detected out of 17 potential off-target sequences). This rate is in the same range as if the potential off-target sequence has only one mismatch located at the position five to eight (35%). In case both mismatches are located at position 9–12, 13–16, or 17–21, off-target effects were detected in in a range up to 52% of the analyzed potential off-target sequences. In case the two mismatches were more distant from each other, off-target effects occurred less frequent. Independently of the position of the mismatches to the PAM, in case the two mismatches were directly next to each other, the off target rate was 38%. In case the mismatches were not located next to each other, the off-target rate was only 15%.

#### Three and More Mismatches Between the Target Sequence and the Potential Off-Target Sequence

Only 0.27% off-target effects (16 out of the 6,028) were detected when there were three or more mismatches. Seven of these sequences had one mismatch between position one to eight proximal to the PAM and the remaining ones beyond position eight. In nine potential off-target sequences, all mismatches were located beyond position eight.

#### Conclusion for Hypothesis 2

The data base (i.e., number of cases per class) for one and two mismatches is relatively limited, but based on the available evidence, there is a tendency that off-target effects are reduced when the mismatch/es are located within the first eight nucleotides proximal to the PAM. If the mismatch/es are located within this region, off-target effects appear reduced compared to the case when the mismatch/es are located outside the seed sequence. Nevertheless, the database regarding one mismatch indicates, that even when the mismatch is located inside this region off-target effects still occurred in around one third of the analyzed potential off-target sequences (compared to 59% observed for the whole guide sequence in Figure 3). It is further indicated that adjoined mismatches increase the likelihood of off-target effects. Hence, care should be taken when selecting a target sequence that the mismatches of potential off-target sequences are not located next to each other.

### Hypothesis 3: GC-Content

**Hypothesis 3**: A lower *guanine-cytosine (GC)-content of the target sequence decreases the occurrence of off-target effects*.

Some scientists suggested that the GC-content of a chosen target sequence may influence off-target effects, as a high GC-content stabilize the sRNA/genomic DNA hybridization (Fu et al., 2013). Therefore, it is estimated that a low GC-content decreases the occurrence of off-target effects (Yu et al., 2017). This hypothesis aims to assess whether the GC-content affects the occurrence of off-target effects. The GC-content was calculated as follows: GC = #{G or C in guide}/guide_length.

#### One Mismatch Between the Target Sequence and the Potential Off-Target Sequence

**Figure 6:**
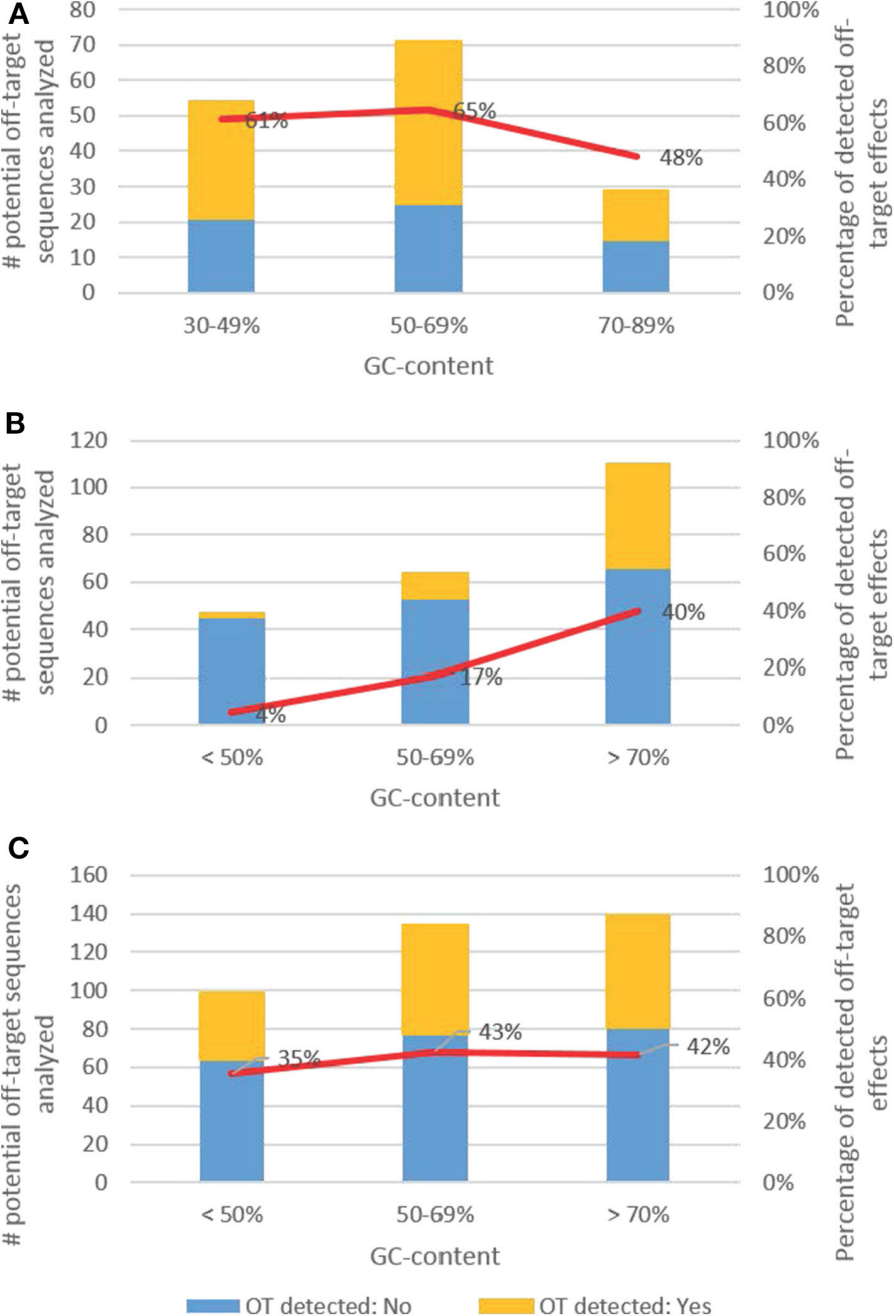
Rate of identified off-target effects assessing potential off-target sequences with **(A)** one mismatch **(B)** two mismatches **(C)** one and two mismatches to the target sequence considering the GC-content of the protospacer.

Figure 6A considers the number of analyzed potential off-target sequences having one mismatch to the target sequence in relation to the GC-contents of the target sequence. The target sequences had a GC-content in a range of 30 and 90%. Due to the limited database, we subdivided the potential off-target sequences in three groups based on the GC-content: 30–49, 50–69, and 70–89%. In case the GC-content was between 30 and 49% (*n* = 54 analyzed potential off-target sequences), 60% of the analyzed off-target sequences showed off-target effects. A similar off-target rate was detected when the GC-content was between 50 and 69%, as 65% of the analyzed sequences showed off-target effects (*n* = 71). If the GC-content was between 70 and 89% (*n* = 29), off-target effects were detected at 48% of the analyzed sequences.

#### Two Mismatches Between the Target Sequence and the Potential Off-Target Sequences

Regarding two mismatches, the GC-content of the target sequences varied in a range of 25 to 95%, but only two sequences had a GC-content below 30% and two higher than 90%. Based on these findings we subdivided the potential off-target sequences in three groups based on the GC-content: <49, 50–69, and >70%. As shown in Figure 6B the number of identified off-target effects increased from 4% identified off-target effects for a GC-content lower than 49% (*n* = 45 analyzed sequences), over 17% when the GC-content was between 50 and 69% (*n* = 53), to 40% in case the GC-content was higher than 70% (*n* = 110).

Regarding one mismatch, the results indicate that the occurrence of off-target effects is decreased when the GC-content is ≥70%, whereas when considering two mismatches, it seems that the off-target rate is increasing when the GC-content is ≥70%. These results may indicate an interaction with the number and location of mismatches, though it cannot be resolved from the given data set. In Figure 6C, we assessed off-target effects for different GC-contents of the target sequence for one and two mismatches combined.

#### Three and More Mismatches Between the Target Sequence and the Potential Off-Target Sequence

Regarding three mismatches, in total 11 off-target effects were identified. In four cases the targeted sequence had a GC-content lower than 50%. Four off-target effects were detected when the target sequence had a GC-content between 50 and 69% and in three cases the target sequence had a GC-content higher than 70%. Regarding at least four mismatches five off-target effects were identified and three of these sequences had a GC-content lower than 50%, while two ones had a GC-content between 50 and 69% (details are provided in Supplementary Table 4).

#### Conclusion for Hypothesis 3

Based on the available evidence the results do not indicate that the occurrence of off-target effects shows an obvious trend in relation to the GC-content of the protospacer sequence. Due to the limited database it was not possible to analyze in detail the GC-content independently of further factors potentially affecting off-target effects.

### Hypothesis 4: Nuclease Variant

**Hypothesis 4**: *Improved nuclease variants decrease the occurrence of off-target effects*.

Much effort is spend into the research of new nucleases and the further development of existing ones. Off-target effects may be reduced due to longer recognition sequences (e.g., SaCas9), different PAMs (e.g., Cpf1) or protein-engineered approaches resulting in altered SpCas9 variants (e.g., SpCas9-HF). Hypothesis 4 aims to investigate off-target effects in different nuclease variants.

Table 8 provides an overview about the analyzed potential off-target sequences for different nuclease variants. In total, 13 different nucleases were investigated. Six thousand two hundred and nineteen potential off-target sequences were analyzed using the standard SpCas9 nuclease (97% of the total). Further 197 potential off-target sequences were analyzed with regards to the nuclease variants Cpf1 (1%), eSpCas9 (1.1) (0.5%), eSpCas9 (1.0) (0.5%), and SaCas9 (0.1%). Another 61 potential off-target sequences (1% of the total) were analyzed for nucleases like eSaCas9, Cas9-HF1, VQR variants, xCas9 3.7, SpCas9-DE, SpCas9-KA, or truncated SpCas9. Since none of these nuclease variants were analyzed in more than three studies their data were summarized among “others.” As the data pool is quite limited except for SpCas9, the direct comparison of the general off-target activity for different nucleases is only rarely possible. The investigated potential off-target sequences of the different nuclease variants differ strongly in the number of mismatches. Regarding the Cpf1 nuclease, only seven potential off-target sequences were examined with one or two mismatches to the target sequences (11%), while the remaining 55 sequences had three or more mismatches. In contrast, the potential off-target sequences of the mutated SpCas9 variants eSpCas9 (1.1) and eSpCas9 (1.0) had one or two mismatches in 91–97% of the investigated sequences, while no sequence had four or more mismatches. Nevertheless, in all nuclease variants off-target effects have been identified if the potential off-target sequence differs from the target one in just one mismatch. Since the data basis is limited for all nucleases with except for the standard nuclease SpCas9, no reliable conclusions can be made about differences in the specificity of different nuclease variants.

**Table 8:** Overview about the characteristics of analyzed potential off-target sequences using different nuclease variants.

**Nuclease variant**	**Number of studies**	**Analyzed potential off-target sequences**	**Number of mismatches off-target sequences**	**Analyzed potential identified**	**Off-target effects**
SpCas9	311	6,219	1 mismatch	95	54
			2 mismatches	147	35
			3 mismatches	344	10
			≥4 mismatches	5,628	5
Cpf1	20	62	1 mismatch	4	3
			2 mismatches	3	0
			3 mismatches	4	0
			≥4 mismatches	51	0
eSpCas9 (1.1)	8	33	1 mismatch	9	6
			2 mismatches	21	9
			3 mismatches	3	1
			≥4 mismatches	0	–
eSpCas9 (1.0)	8	32	1 mismatch	10	8
			2 mismatches	21	8
			3 mismatches	1	0
			≥4 mismatches	0	–
SaCas9	6	9	1 mismatch	4	4
			2 mismatches	1	0
			3 mismatches	0	–
			≥4 mismatches	4	0
Others	17	61	1 mismatch	32	18
			2 mismatches	25	5
			3 mismatches	4	0
			≥4 mismatches	0	–

However, in three articles, targeted experiments were conducted by applying the same study design differing just by the nuclease variant to directly compare their off-target effects. In the first article four different nuclease variants (CRISPR/SpCas9, CRISPR/eSpCas9 (1.0), CRISPR/eSpCas9 (1.1), and CRISPR/SpCas9-HF1) were compared for off-target effects (Zhang et al., 2017). The off-target analysis showed that the three altered SpCas9 variants CRISPR/eSpCas9 (1.0), CRISPR/eSpCas9 (1.1), and CRISPR/SpCas9-HF1 had substantially less off-target effects compared to the wild type SpCas9 variant and SpCas9-HF1 consistently exhibited the lowest off-target activity at the five examined potential off-target sequences. In addition, the on-target: off-target indel frequency ratios of the three altered SpCas9 variants were, on average, 273-fold higher compared to wild type SpCas9. However, no nuclease variant was completely free of off-target effects when there was just one mismatch between the on-target and potential off-target sequence. Raitskin et al. (2019) conducted three experiments. In the first one they directly compared five variants of SpCas9 [CRISPR/SpCas9, CRISPR/SpCas9-DE, CRISPR/SpCas9-KA, CRISPR/eSpCas9 (1.0), CRISPR/eSpCas9 (1.1.)] by designing a set of five sgRNAs each with a mutation in a different base of the spacer. Results indicate that while the number of mutations induced by standard SpCas9 was significantly reduced when the spacer contained a mutation in the region close to the PAM, the presence of a mismatch between the spacer and target in the distal region had minimal effects. In contrast, the frequency of mutations induced by the variants eCas9 1.0 and 1.1 was significantly reduced by a mismatch in any region of the sgRNA (Raitskin et al., 2019). In the second experiment, four nuclease variants [CRISPR/SpCas9, CRISPR/eSpCas9 (1.0), CRISPR/eSpCas9 (1.1), CRISPR/xCas9 3.7] were compared and eight targets were addressed, all having one mismatch to a potential off-target sequence. Wild type SpCas9 induced eight on-target mutations and mutations were detected in all corresponding off-target sequences. Compared to this the SpCas9 variants were only able to induce mutations at three [CRISPR/eSpCas9 (1.0), CRISPR/xCas9 3.7] or four [CRISPR/eSpCas9 (1.1)] on-targets and these variants did not always reduce mutagenesis at the off-target sequences. In the third experiment the efficiency and specificity of SaCas9 was compared with eSaCas9 by addressing four sequences that have a potential off-target sequence with one mismatch. Sa Cas9 induced mutations at all four on-targets as well as the corresponding potential off-target sequences, while eSaCas9 were only able to induced three on-target mutations and only one potential off-target sequence was mutated (Raitskin et al., 2019). In the third study, Endo et al. (2019) compared off-target mutations induced by SpCas9 and SpCas9-NGv1 in rice. The results showed that off-target effects were detected for wild type SpCas9 when there was one mismatch at position 19 proximal to the PAM, while no mutations were detected when the mismatch was located at the 6th nucleotide from the PAM. Compared to this, no off-target effects were detected for SpCas9-NGv1 independently on the position of the mismatch.

#### Conclusion for Hypothesis 4

The data pool for the occurrence of off-target effects when employing the standard nuclease SpCas9 is large. For all other nuclease variants, we identified only a very limited number of analyzed potential off-target sequences. Therefore, no conclusions can be made regarding this hypothesis providing a general tendency. However, a few investigations directly compared different nuclease variants. In these experiments the likelihood of off-target effects was reduced by using alternative nucleases, but off-target effects were not completely excluded when sequences were similar to the target sequence.

### Hypothesis 5: Delivery Method

***Hypothesis 5*:** *The period in which the CRISPR/Cas system is active in the plant cell affects the occurrence of off-target effects*.

A further aspect that may have an impact on the occurrence of off-target effects is the delivery of the CRISPR/Cas system into the plant cell. It can be delivered to the (plant) cell either as DNA (transiently or stably expressed), RNA or directly as Ribonucleoproteins (RNP). Stable transformation leads to a permanent expression of the CRISPR/Cas system compared to the other approaches in which the CRISPR/Cas system is available only for a limited time. Therefore, it is assumed that a permanent expression leads to an increased on-target as well as an increased off-target impact (Zischewski et al., 2017; Metje-Sprink et al., 2018; Jansing et al., 2019). Additionally, DNA-free approaches using RNP or RNA may further reduce off-target effects, as the CRISPR/Cas system is degraded rapidly within the range of few days and the mode of action is only present in the edited cells but not in the regenerated plants (Woo et al., 2015; Metje-Sprink et al., 2018). Hypothesis 5 aims to investigate off-target effects considering the period of time in which CRISPR/Cas9 is active provoked by different delivery methods.

Table 9 provides an overview about the number of analyzed potential off-target sequences for different delivery methods. Three hundred and twenty one studies delivered the CRISPR/Cas system as DNA with stable integration into the genome and subsequent expression (referred as *DNA-stable* in the table). Within these studies, 6,163 potential off-target sequences were analyzed (96% of the total). Thirty nine studies supplied the CRISPR/Cas system as DNA but the system was not integrated into the genome and hence only transiently expressed; in total 111 potential off-target sequences were analyzed (referred as *DNA-transient* in the table). In eight studies, *RNP* were used to deliver directly the nuclease and the RNA and 133 potential off-target sequences were analyzed. In two studies, RNA was used to deliver the CRISPR/Cas system into the plant cell and nine potential off-target sequences were analyzed.

**Table 9:** Overview about the characteristics of analyzed potential off-target sequences using delivery methods to deliver the CRISPR system into the plant cells.

**Nuclease variant**	**Number of studies**	**Analyzed potential off-target sequences**	**Number of mismatches**	**Analyzed potential off-target sequences**	**Off-target effects identified**
DNA-stable	321	6,163	1 mismatch	101	52
			2 mismatches	207	55
			3 mismatches	323	9
			≥4 mismatches	5,532	4
DNA-transient	39	111	1 mismatch	51	39
			2 mismatches	7	1
			3 mismatches	18	2
			≥4 mismatches	35	1
RNP	8	133	1 mismatch	1	1
			2 mismatches	4	1
			3 mismatches	13	0
			≥4 mismatches	115	0
RNA	2	9	1 mismatch	1	1
			2 mismatches	0	–
			3 mismatches	2	0
			≥4 mismatches	6	0

*RNP, Ribonucleoproteins; DNA, deoxyribonucleic acid; RNA, ribonucleic acid*.

The off-target rate for the *DNA-stable* approach and *RNP* appear around 2%, which is much lower than for the *DNA-transient* approach with 39%. But, examining the data in detail also demonstrate that the data for the different delivery methods represent considerable systematic differences in the likelihood that off-target effects can be observed: following from the results derived for hypothesis 1 the uneven distribution of low and high number of mismatches between the delivery methods counteract a balanced evaluation. Applying the *DNA-stable* approach, only 5% of the investigated potential off-target sequences represent one or two mismatches to the target sequence (with higher likelihood of off target effects) while for the *DNA-transient* approach 52% of the off-target sequences investigated referred to one or two mismatches. This unequal distribution of the numbers of mismatches per sequence in the analyzed samples is as well-pronounced for the investigation conducted with *RNP*. Only 4% off-target sequences differed from the target sequence in one or two mismatches, while 96% had at least three mismatches. Due to the low comparability of included studies, no sound conclusions can be drawn based on the general overview of all studies.

Three targeted experiments were conducted by applying the same study design, differing just by the delivery method to directly compare off-target effects. Two of these articles were published by one institution and delivery methods (DNA-stable, DNA-transient, RNP, RNA) were applied and compared in hexaploid bread wheat (Zhang et al., 2016; Liang et al., 2017). The authors edited a target sequence of the TaGW2 gene, which shows identical sequences on the chromosomes B and D, whereas on chromosome A it has one mismatch at position nine proximal to the PAM. Independently of the applied delivery methods, an off-target effect occurred at this potential off-target sequence. However, Liang et al. reported that RNP showed a decreased on-target and off-target mutation frequency compared to the DNA-transient approach [RNP: Mutagenesis frequencies for TaGW2-B1 and -D1 (on-target): 33.4 and 21.8%, for TaGW2-A1 (off-target): 5.7%; DNA-transient: Mutagenesis frequencies for TaGW2-B1 and -D1 (on-target): 42.2 and 35.6%, for TaGW2-A1 (off-target): 30.8%] (Liang et al., 2017). Zhang et al. reported that delivering the system as RNA, the on- and off-target mutation frequency decreased compared to DNA-stable and DNA-transient delivery approach (RNA: Mutagenesis frequencies for TaGW2-B1 and –D1 (on-target): 1.1 and 1.1%, for TaGW2-A1 (off-target): 0.4%; DNA-stable: Mutagenesis frequencies for TaGW2-B1 and -D1 (on-target): 2.9 and 2.6%, for TaGW2-A1 (off-target): 2.0%; DNA-transient: Mutagenesis frequencies for TaGW2-B1 and -D1 (on-target): 2.9 and 3.0%, for TaGW2-A1 (off-target): 2.3%) (Zhang et al., 2016). In addition, in this study, further potential off-target sequences were predicted and analyzed. Eight potential off-target sequences with three to four mismatches to the target sequence were analyzed for the DNA-stable, DNA-transient and RNA approach but none of these showed the occurrence of off-target effects (Zhang et al., 2016). In another article, Svitashev et al. (2016)) conducted a DNA-stable and RNP delivery experiment by addressing the MS45 gene in rice (Svitashev et al., 2016). This site had one potential off-target sequence with two mismatches at position 12 and 20 proximal to the PAM. In both experiments off-target effects were detected, but the RNP off-target mutation rate was decreased compared to the DNA-stable delivery approach [DNA-stable: Mutagenesis frequency for MS45 (on-target): 0.34%, MS45 (off-target sequence): 0.18%; RNP: Mutagenesis frequency for MS45 (on-target): 0.69%, MS45 (off-target sequence): 0.01%].

#### Conclusion for Hypothesis 5

The data pool to assess this hypothesis for the occurrence of off-target effects when applying the *DNA-stable* approach is large, while for *DNA-transient, RNP* and *RNA* only a limited number of analyzed potential off-target sequences exist. Therefore, based on the available evidence it is not possible to conclude on this hypothesis. However, direct comparison of different delivery methods indicate that the off-target rate can be reduced (but not totally avoided) by delivering the CRISPR/Cas system to the plant cell either as *DNA-transient, RNA* or *RNP*.

### Descriptive Synthesis of Low and Medium/Low as Well as Unclear Validity Studies

#### Studies of Low and Medium/Low Validity

In total, 72 studies were rated as having *low validity* and 44 as having *medium/low validity*. The reason for rating as low or *medium/low validity* is summarized in Table 5. Detailed information about the validity assessment of all studies including the reason for the classification is provided in Supplementary Table 5. Twenty seven studies were rated as *low validity* as the CRISPR/Cas technique did not induce an on-target mutation. Within these studies, three off-target effects were detected in three different studies. Two of these studies had additional differences between the genotype and the reference genome that may explain off-target effects. In one study no on-target mutation was detected but a potential off-target sequence with one mismatch at position 20 proximal to the PAM showed an off-target effect. In seven studies, no reference genome has been fully sequenced or the authors remark that sequencing errors may be responsible for off-target effects. Within these studies, four off-target effects were detected in three different studies. In 15 studies, no PAM followed the analyzed potential off-target sequences. No mutations were detected when assessing potential off-target effects in these studies. In four studies, the identified so called “off-target sequences” were actually identical to the on-target sequence. In three of these studies, mutations were detected. Twenty three studies didn't provide any information about the analyzed potential off-target sequences, and in none of these studies off-target effects were reported. In one study, the sgRNA/Cas9 complex was extremely overexpressed but no information were provided how this was achieved. Within this study, 10 potential off-target sequences were analyzed and eight off-target effects have been identified. All of these sequences had three or four mismatches to the target sequence.

Regarding studies rated as having *medium/low validity*, in 28 studies potential off-target sequences were assessed, but not all of the analyzed potential off-target sequences were followed by a PAM. Potential off-target sequences without a PAM were selected as *medium/low validity* and no off-target effects had been detected in any of these sequences. Fifteen studies used Whole Genome Sequencing but information about potential off-target sequences were incomplete. None of these potential off-target sequences with incomplete information were documented showing off-target effects. In seven studies, some named off-target sequences were actually identical to the on-target sequence and one off-target mutation was detected.

#### Studies of Unclear Validity

Corresponding authors of studies that lack sufficient information on study conduct were asked to provide missing information but for 26 studies we did not get any response. In 18 studies, potential off-target sequences were analyzed but no information was provided whether a PAM followed the potential off-target sequences. Within these studies, two off-target effects were detected. One off-target effect had one mismatch at position nine proximal to the PAM and the other one had two mismatches at position eight and 20 proximal to the PAM. Eight studies were rated as *unclear validity* as the provided information about potential off-target sequences were convoluted. In five studies, the potential off-target sequence could not be assigned to any target sequence. Another three studies focused on the first 12 nucleotides proximal to the PAM only, while no information about the nucleotides 13 to 20 have been provided. For details see Supplementary Table 5.

## Discussion

### Factors Affecting Off-Target Effects Caused by the Use of CRISPR/Cas in Plants

This systematic review aimed to collect and synthesize the available evidence about factors that may affect the occurrence of off-target effects caused by the application of CRISPR/Cas-system in plants. The 370 studies rated as *high* or *medium/high validity* assessed a total amount of 6,416 potential off-target sequences indicating a robust evidence base. However, only 154 analyzed potential off-target sequences showed one mismatch (2.4% of the total analyzed sequences) and 218 had two mismatches (3.4% of the total analyzed sequences), while the remaining 6,044 sequences (94.2%) referred to three or more mismatches. The reason for this may be that it is frequently recommended to select target sequences with at least three mismatches to other similar sequences and in many articles this criterion has been taken into account when choosing a target sequence (e.g., Collonnier et al., 2017; Martín-Pizarro et al., 2019). The fact that there are several studies in which potential off-target sequences with one or two mismatches have been investigated may have two reasons:

- A target sequence was deliberately chosen with other very similar sequences in the genome in order to further investigate the occurrence of off-target effects.
- Polyploidic plant species were edited that have highly similar homoeoalleles with only one nucleotide mismatch. In these cases off-target effects can be a benefit by increasing the multiplexing capacity (Jacobs et al., 2017).

The evidence base was well-suited to confirm hypothesis 1 that an increase number of mismatches between the on-target sequence and the potential off-target sequence steeply decreases the likelihood that off-target effects occur. Descriptive and quantitative analysis clearly indicate that there is a significant difference in the occurrence of off-target effects for one, two, three, and at least four mismatches. Within this pool of data, the off-target rate (Number of detected off-target effects/number of analyzed potential off-target sequences) for one mismatch appeared to be nearly 60% of the analyzed sequences, while nearly no off-target effect occurs for a sequence with at least four mismatches to further similar sequences in the genome. These findings are in line with several articles published so far (e.g., Fu et al., 2013; Hahn and Nekrasov, 2018; Doll et al., 2019). The observation that a single mismatch between on- and off-target sequences often leads to off-target effects while designing a highly specific sgRNA reduces off-target effects to a minimum allows a flexible choice of sgRNA design depending on the research question. This flexibility can be used as benefit either to mutate several similar alleles in a single experiment or to design a highly specific sgRNA which possesses four or more mismatched positions to all other sequences in the genome.

Potential off-target sequences with three or more mismatches were not suitable to investigate hypotheses 2–5 in detail, as independently on the position of mismatches proximal to the PAM, the GC-content of the protospacer, the used nuclease variants and the applied delivery methods, nearly no off-target effects occurred. Therefore, hypotheses 2–5 were descriptively investigated for one and two mismatches separately comprising a total evidence base of only 352 analyzed potential off-target sequences. Although, the data base for one and two mismatches is limited, based on the available evidence there is a tendency that off-target effects are reduced when the mismatch/es are located within the first eight nucleotides proximal to the PAM. Statistical meta-analysis also indicates that the position of the mismatch/es significantly affects the occurrence of off-target effects but less intense compared to the number of mismatches of the on-target and off-target sequences. These results are consistent with previous findings (e.g., Mali et al., 2013; Endo et al., 2015; Jiang et al., 2015). However, the data base is fairly limited to define properly the number of nucleotides that form the “seed sequence”; but based on the reviewed data we suggest to define it to be eight nucleotides proximal to the PAM. If the mismatch/es are located within this region off-target effects are reduced compared to when the mismatch/es are located outside the seed sequence. The data pool for one mismatch indicates that even if the mismatch is located inside the defined seed sequence, off-target effects may still occur in about one third of the potential off-target sequences which, nevertheless, is half the rate of cases compared with a mismatch at any position of the target sequence. The data pool for two mismatches further indicates that care should be taken when selecting a target sequence for genome editing with at least two potential mismatches that these are not located next to each other; but further research is needed to address this aspect more thoroughly. There was no clear indication found that the GC-content of the target sequence significantly affects the occurrence of off-target effects. In one article it was argued that the low GC-content of the target sequence (45%) is the reason that no off-target effects occurred (Yu et al., 2017), but actually the potential off-target sequences showed four and seven mismatch to the target sequence which could be sufficient to reduce the likelihood to zero according to the conclusion drawn for hypothesis 1. Regarding the nuclease variants and the delivery methods the given data base is considerably poor as the large majority of studies applied the standard nuclease SpCas9 and a cassette for the CRISPR/Cas system were stably integrated to the genome. The available evidence does not allow to conclude that altered nuclease variants or delivery methods significantly reduce the occurrence of off-target effects in general. However, a limited number of articles directly compare different delivery methods or nuclease variants indicate that such different approaches might decrease the occurrence of off-target effects but cannot completely preclude it.

In the given set of data it is likely that various factors have overlapping impacts. Either different intensities and/or insufficient data may have limited their quantitative assessment. This review highlighted the number of mismatches and the occurrence of mismatches in the seed sequence as significant impact factors determining off-target effects of the CRISPR/Cas-System in plants in a general overview of published scientific studies.

### Reasons for Heterogeneity

Besides the limited evidence base to answer hypotheses 2–5 in more detail, the individual studies differ widely in design and conduct. Considering all analyzed potential off-target sequences with one or two mismatches, we identified heterogeneity between studies regarding:

- The position of mismatches proximal to the PAM: Nucleotide mismatches occurred in 21 different positions proximal to the PAM.
- The GC-content of the protospacer: GC-content varied widely between 25 and 95%.
- The used nuclease variants: In total, 13 different nuclease variants were used.
- The applied delivery method: The CRISPR/Cas system was supplied to the plant cell either as DNA (stably or transiently expressed), RNA or directly as RNP, consequently leading to different amounts of protein and varying exposure times of the protein to the target DNA.

Beside to these factors that were assessed within this systematic review, additional characteristics further increased heterogeneity: In total, 43 different plant species were used for the application of CRISPR/Cas. In principle, plants with bigger genomes and higher ploidy levels have statistically more similar sequences to the target sequence in the genome than those with small genomes and a diploid set of chromosomes. In most studies a biased detection method approach was applied to identify potential off-target sequences, meaning that only one or a few similar sequences were identified a priori and only these sequences were assessed for the detection of off-target effects. However, this approach is not suitable for assessing the influence of the genome size of different plant species on off-target effects. The ploidy level may have a stronger influence on the occurrence of off-target effects as polyploidic plants may have very similar homoeoalleles with only a single nucleotide mismatch. Editing one of such sequences may increase the finding of off-target effects. However, as it is often intended to edit all homoeoalleles scientists may look for desired off-target cutting (Wang et al., 2014; Liang et al., 2017). In addition, many different bioinformatics tools and detection methods have been used to predict potential off-target sequences and to investigate them for off-target effects.

### Review Limitations

#### Limitations of the Review Methodology

The search for relevant literature was limited to German and English language, whereby all identified relevant literature was in English. Therefore, we might have missed studies published in other languages. More than half of the relevant studies were conducted in an Asian country and literature was also identified in Asian languages but excluded on title/abstract level due to language barriers. It can be assumed that a literature search in these languages would identify further relevant studies. In addition, no studies have been identified with corresponding authors from South America, although it is known that genome editing is already broadly used there. A search in Spanish or Portuguese would possibly also identify additional relevant literature. Nevertheless, a detailed evaluation of each author's role and locations which would provide more details about any international cooperation was not in the scope of this review. The full text of 49 articles that have been rated as relevant on title/abstract level were not accessible within the course of the review project and therefore they were not included in this systematic review (see Supplementary Table 2).

#### Limitations of the Evidence Base

Altogether the evidence base is comprehensive with over 6,400 analyzed potential off-target sequences. This was sufficient to evaluate and identify some general key factors which determine the occurrence of off-target effects. However, more than 94% of the analyzed sequences represented cases with three or more mismatches to the target sequence. Since off-target effects rarely occurred in these sequences, the evidence base was not adequate to allow clear conclusions about all but two factors potentially affecting the occurrence of off-target effects. In addition, the available studies were very heterogeneously designed. Therefore, the assessment of the hypotheses regarding the roles of the GC-content, the nuclease variant and the delivery method were restricted and no concluding evaluation can be made for those factors. This identified evidence gap needs to be filled by systematic studies that apply the same study design varying just the specific form of one impact factor to be tested (e.g., the delivery method or the nuclease variant). For these experiments, one should use an almost identical reference sequence in several different plant species to allow a more generalizing evaluation of their potential to affect the occurrence of off-target effects. In addition, more experiments should be done designing a set of sgRNAs for a specific target each with a mutation in a different base of the whole sgRNA (similar ones have been done in (e.g., Zhang et al., 2017; Raitskin et al., 2019). Doing so, it is for example possible to address the weight of each sgRNA position for on- and off-target activity and in parallel to evaluate the importance of proper folding of the HNH domain as well. It might came out that for a given target a less perfect sgRNA can achieve even higher on-target rates due to better HNH folding or tighter DNA:RNA binding.

## Conclusions

### Implication for Policy/Management

The risk of off-target effects in plants caused by genome editing approaches as by the broadly employed CRISPR/Cas system is not as critical as in clinical research. As for the latter side effects have to be excluded to protect future patients, for plants the identified off-target mutations can be segregated by outcrossing or mutants without off-target effects can be selected for further variety development (Zhang et al., 2016). Since plant breeding is the immediate context in this review, it is worth to recall the occurrence of off-target effects in natural mutations and routinely used breeding techniques such as regular crossing or undirected mutagenesis using tissue cultures, chemical mutagens or irradiation (Scientific Advice Mechanism (SAM), 2017). The natural mutation rate of Arabidopsis thaliana is approximately one mutation per 150,000 kilobase pairs (kbp) which means that around one mutation occurs per generation (Ossowski et al., 2010; Bartsch et al., 2018). Using chemicals like EMS or irradiation to induce mutations in plants the mutation rate increases dramatically, e.g., Jander et al. identified at least 700 mutations in EMS-mutagenized Arabidopsis lines (Jander et al., 2003). Another example is the regeneration of plants from cell culture (somaclonal variation). Experiments showed that somaclonal variation increases the mutation rate by a factor up to 250 compared to spontaneous mutations (Miyao et al., 2012). Compared to these techniques, off-target effects through genome-editing occur by orders of magnitude less frequently. Risk assessors and decision makers should take this aspect into account when drawing conclusions about general risks being associated with the application of genome editing in plants.

### Implication for Research

Around 10% of the initially identified potentially relevant studies could not be considered in this review due to lacking information about the study design and/or the off-target sequences. To enable broader analysis and evaluation the following information should be provided when analyzing off-target effects:

- The reference on-target sequence.
- The prediction tools and detection methods used.
- and for the potential off-target sequences:
  - The number of identified potential off-target sequences.
  - The sequences of the potential off-target sites plus the PAM (and therewith the number and the position of the mismatches proximal to the PAM).

The results of the review show that the occurrence of off-target effects prominently depends on the number of mismatches to other similar sequences and the position of the mismatch/es proximal to the PAM. So far, only a few studies applied an identical study design by systematically varying the modifications of one impact factor, in order to examine the impacts on the occurrence of off-target effects. More comparative studies are necessary to provide oversight of a general impact pattern to guide further application in research and development.

In order to minimize off-target effects *a priori*, it is recommended from the analysis of hypothesis 1 and 2 that a target sequence is chosen which differs in at least four disjunct positions from similar genomic sequences. This can reduce further crossing or selection efforts to support thorough investigations of e.g., gene functions or variety development.

## Data Availability Statement

The original contributions presented in the study are included in the article/Supplementary Materials, further inquiries can be directed to the corresponding author/s.

## Author Contributions

The review process was coordinated by DM. DM, FH, and TS conducted the screening of articles. DM extracted metadata and drafted the report. HL carried out statistical analysis. All authors participated in the critical appraisal of studies and assisted in editing and revising the final manuscript.

## Conflict of Interest

The authors declare that the research was conducted in the absence of any commercial or financial relationships that could be construed as a potential conflict of interest.

## References

[B1] Altschul S. (1990). Basic local alignment search tool. J. Mol. Biol. 215, 403–410. 10.1016/S0022-2836(05)80360-2 2231712

[B2] Bartsch D. Bendiek J. Braeuning A. Dagand E. Duensing N. Fladung M. (2018). Wissenschaftlicher Bericht zu den Neuen Techniken in der Pflanzenzüchtung und der Tierzucht und Ihren Verwendungen im Bereich der Ernährung und Landwirtschaft. Available online at: https://www.bmel.de/SharedDocs/Downloads/DE/_Landwirtschaft/Gruene-Gentechnik/Bericht_Neue_Zuechtungstechniken.html (accessed February 23, 2018).

[B3] Bertier L. D. Ron M. Huo H. Bradford K. J. Britt A. B. Michelmore R. W. (2018). High-resolution analysis of the efficiency, heritability, and editing outcomes of CRISPR/Cas9-induced modifications of NCED4 in Lettuce (*Lactuca sativa*). G3 8, 1513–1521. 10.1534/g3.117.300396 29511025 PMC5940144

[B4] Chen J. S. Dagdas Y. S. Kleinstiver B. P. Welch M. M. Harrington L. B. Sternberg S. H.. (2017). Enhanced proofreading governs CRISPR-Cas9 targeting accuracy. Nature 550, 407–410. 10.1038/nature24268 28931002 PMC5918688

[B5] Collaboration for Environmental Evidence (2018). Guidelines and Standards for Evidence Synthesis in Environmental Management. Version 5.0, eds Pullin A. S. Frampton G. K. Livoreil B. Petrokofsky G. Available online at: http://www.environmentalevidence.org/guidelines/table-of-contents (accessed November 3, 2019).

[B6] Collonnier C. Epert A. Mara K. Maclot F. Guyon-Debast A. Charlot F.. (2017). CRISPR-Cas9-mediated efficient directed mutagenesis and RAD51-dependent and RAD51-independent gene targeting in the moss *Physcomitrella patens*. Plant Biotechnol. J. 15, 122–131. 10.1111/pbi.12596 27368642 PMC5253467

[B7] Dagdas Y. S. Chen J. S. Sternberg S. H. Doudna J. A. Yildiz A. (2017). A conformational checkpoint between DNA binding and cleavage by CRISPR-Cas9. Science Adv. 3:eaao0027. 10.1101/122242 28808686 PMC5547770

[B8] Doll N. M. Gilles L. M. Gérentes M. F. Richard C. Just J. Fierlej Y.. (2019). Single and multiple gene knockouts by CRISPR–Cas9 in maize. Plant Cell Rep. 38, 487–501. 10.1007/s00299-019-02378-1 30684023

[B9] Dönmez D. Simsek Ö. Aka Kacar Y. (2016). Genetic engineering techniques in fruit science. IJOEAR. 2, 115–128.

[B10] Eckerstorfer M. F. Dolezel M. Heissenberger A. Miklau M. Reichenbecher W. Steinbrecher R. A. (2019a). An EU perspective on biosafety considerations for plants developed by genome editing and other new genetic modification techniques (nGMs). Front. Bioeng. Biotechnol. 7:31 10.3389/fbioe.2019.00090 30891445 PMC6413072

[B11] Eckerstorfer M. F. Engelhard M. Heissenberger A. Simon S. Teichmann H. (2019b). Plants developed by new genetic modification techniques-comparison of existing regulatory frameworks in the EU and non-EU countries. Front. Bioeng. Biotechnol. 7:26. 10.3389/fbioe.2019.00026 30838207 PMC6389621

[B12] Endo M. Mikami M. Endo A. Kaya H. Itoh T. Nishimasu H.. (2019). Genome editing in plants by engineered CRISPR–Cas9 recognizing NG PAM. NPLANTS. 5, 14–17. 10.1038/s41477-018-0321-8 30531939

[B13] Endo M. Mikami M. Toki S. (2015). Multigene knockout utilizing off-target mutations of the CRISPR/Cas9 system in rice. Plant Cell Physiol. 56, 41–47. 10.1093/pcp/pcu154 25392068 PMC4301742

[B14] Fu Y. Foden J. A. Khayter C. Maeder M. L. Reyon D. Joung J. K.. (2013). High-frequency off-target mutagenesis induced by CRISPR-Cas nucleases in human cells. Nat. Biotechnol. 31, 822–826. 10.1038/nbt.2623 23792628 PMC3773023

[B15] Haddaway N. R. Macura B. Whaley P. Pullin A. S. (2018). ROSES RepOrting standards for systematic evidence syntheses: pro forma, flow-diagram and descriptive summary of the plan and conduct of environmental systematic reviews and systematic maps. Environ Evid. 7:409 10.1186/s13750-018-0121-7

[B16] Hahn F. Nekrasov V. (2018). CRISPR/Cas precision: do we need to worry about off-targeting in plants? Plant Cell Rep. 38, 437–441. 10.1007/s00299-018-2355-9 30426198 PMC6469637

[B17] HinzJ. M.LaugheryM. F.WyrickJ. J. (2015). Nucleosomes inhibit Cas9 endonuclease activity *in vitro*. Biochemistry 54, 7063–7066. 10.1021/acs.biochem.5b0110826579937

[B18] Hsu P. D. Scott D. A. Weinstein J. A. Ran F. A. Konermann S. Agarwala V.. (2013). DNA targeting specificity of RNA-guided Cas9 nucleases. Nat. Biotechnol. 31, 827–832. 10.1038/nbt.2647 23873081 PMC3969858

[B19] Jacobs T. B. LaFayette P. R. Schmitz R. J. Parrott W. A. (2015). Targeted genome modifications in soybean with CRISPR/Cas9. BMC Biotechnol. 15:16. 10.1186/s12896-015-0131-2 25879861 PMC4365529

[B20] Jacobs T. B. Zhang N. Patel D. Martin G. B. (2017). Generation of a collection of mutant tomato lines using pooled CRISPR libraries. Plant Physiol. 174, 2023–2037. 10.1104/pp.17.00489 28646085 PMC5543939

[B21] Jander G. Baerson S. R. Hudak J. A. Gonzalez K. A. Gruys K. J. Last R. L. (2003). Ethylmethanesulfonate saturation mutagenesis in Arabidopsis to determine frequency of herbicide resistance. Plant Physiol. 131, 139–146. 10.1104/pp.102.010397 12529522 PMC166794

[B22] Jansing J. Schiermeyer A. Schillberg S. Fischer R. Bortesi L. (2019). Genome editing in agriculture: technical and practical considerations. Int. J. Mol. Sci. 20:2888. 10.3390/ijms20122888 31200517 PMC6627516

[B23] Ji X. Si X. Zhang Y. Zhang H. Zhang F. Gao C. (2018). Conferring DNA virus resistance with high specificity in plants using virus-inducible genome-editing system. Genome Biol. 19:197. 10.1186/s13059-018-1580-4 30442181 PMC6238286

[B24] Jiang F. Zhou K. Ma L. Gressel S. Doudna J. A. (2015). Structural biology. a Cas9-guide RNA complex preorganized for target DNA recognition. Science 348, 1477–1481. 10.1126/science.aab1452 26113724

[B25] Jiang W. Z. Henry I. M. Lynagh P. G. Comai L. Cahoon E. B. Weeks D. P. (2017). Significant enhancement of fatty acid composition in seeds of the allohexaploid, *Camelina sativa*, using CRISPR/Cas9 gene editing. Plant Biotechnol. J. 15, 648–657. 10.1111/pbi.12663 27862889 PMC5399004

[B26] JMP® (2019). JMP®. Version 14. Cary, NC: SAS Institute Inc.

[B27] Kaya H. Mikami M. Endo A. Endo M. Toki S. (2016). Highly specific targeted mutagenesis in plants using *Staphylococcus aureus* Cas9. Sci. Rep. 6, 1–9. 10.1038/srep26871 27226350 PMC4881040

[B28] Kohl C. McIntosh E. J. Unger S. Haddaway N. R. Kecke S. Schiemann J. (2018). Online tools supporting the conduct and reporting of systematic reviews and systematic maps: a case study on CADIMA and review of existing tools. Environ Evid. 7:2420 10.1186/s13750-018-0124-4

[B29] LeBlanc C. Zhang F. Mendez J. Lozano Y. Chatpar K. Irish V. F.. (2018). Increased efficiency of targeted mutagenesis by CRISPR/Cas9 in plants using heat stress. Plant J. 93, 377–386. 10.1111/tpj.13782 29161464

[B30] Lee J. Chung J.-H. Kim H. M. Kim D.-W. Kim H. (2016). Designed nucleases for targeted genome editing. Plant Biotechnol. J. 14, 448–462. 10.1111/pbi.12465 26369767 PMC11389202

[B31] Lei Y. Lu L. Liu H.-Y. Li S. Xing F. Chen L.-L. (2014). CRISPR-P: a web tool for synthetic single-guide RNA design of CRISPR-system in plants. Mol. Plant 7, 1494–1496. 10.1093/mp/ssu044 24719468

[B32] Liang Z. Chen K. Li T. Zhang Y. Wang Y. Zhao Q.. (2017). Efficient DNA-free genome editing of bread wheat using CRISPR/Cas9 ribonucleoprotein complexes. Nat. Commun. 8:14261. 10.1038/ncomms14261 28098143 PMC5253684

[B33] Liu G. Yin K. Zhang Q. Gao C. Qiu J.-L. (2019). Modulating chromatin accessibility by transactivation and targeting proximal dsgRNAs enhances Cas9 editing efficiency *in vivo*. Genome Biol. 20:145. 10.1186/s13059-019-1762-8 31349852 PMC6660936

[B34] Liu H. Ding Y. Zhou Y. Jin W. Xie K. Chen L.-L. (2017). CRISPR-P 2.0: an improved CRISPR-cas9 tool for genome editing in plants. Mol. Plant 10, 530–532. 10.1016/j.molp.2017.01.003 28089950

[B35] Mali P. Aach J. Stranges P. B. Esvelt K. M. Moosburner M. Kosuri S.. (2013). CAS9 transcriptional activators for target specificity screening and paired nickases for cooperative genome engineering. Nat. Biotechnol. 31, 833–838. 10.1038/nbt.2675 23907171 PMC3818127

[B36] Martin F. Sánchez-Hernández S. Gutiérrez-Guerrero A. Pinedo-Gomez J. Benabdellah K. (2016). Biased and unbiased methods for the detection of off-target cleavage by CRISPR/Cas9: an overview. Int. J. Mol. Sci. 17:1507. 10.3390/ijms17091507 27618019 PMC5037784

[B37] Martín-Pizarro C. Triviño J. C. Posé D. (2019). Functional analysis of the TM6 MADS-box gene in the octoploid strawberry by CRISPR/Cas9-directed mutagenesis. J. Exp. Bot. 70, 949–961. 10.1093/jxb/ery400 30428077 PMC6363087

[B38] Metje-Sprink J. Menz J. Modrzejewski D. Sprink T. (2018). DNA-free genome editing: past, present and future. Front. Plant Sci. 9:1957. 10.3389/fpls.2018.01957 30693009 PMC6339908

[B39] Mitchell B. P. Hsu R. V. Medrano M. A. Zewde N. T. Narkhede Y. B. Palermo G. (2020). Spontaneous embedding of DNA mismatches within the RNA:DNA hybrid of CRISPR-Cas9. Front. Mol. Biosci. 7:39. 10.3389/fmolb.2020.00039 32258048 PMC7093078

[B40] Miyao A. Nakagome M. Ohnuma T. Yamagata H. Kanamori H. Katayose Y.. (2012). Molecular spectrum of somaclonal variation in regenerated rice revealed by whole-genome sequencing. Plant Cell Physiol. 53, 256–264. 10.1093/pcp/pcr172 22156226

[B41] Modrzejewski D. Hartung F. Sprink T. Krause D. Kohl C. Wilhelm R. (2019). What is the available evidence for the range of applications of genome-editing as a new tool for plant trait modification and the potential occurrence of associated off-target effects: a systematic map. Environ Evid. 8:11 10.1186/s13750-019-0171-5

[B42] Montague T. G. Cruz J. M. Gagnon J. A. Church G. M. Valen E. (2014). CHOPCHOP: a CRISPR/Cas9 and TALEN web tool for genome editing. Nucleic Acids Res. 42, W401–W407. 10.1093/nar/gku410 24861617 PMC4086086

[B43] Ossowski S. Schneeberger K. Lucas-Lled,ó J. I. Warthmann N. Clark R. M. Shaw R. G.. (2010). The rate and molecular spectrum of spontaneous mutations in *Arabidopsis thaliana*. Science 327, 92–94. 10.1126/science.1180677 20044577 PMC3878865

[B44] Puchta H. Fauser F. (2014). Synthetic nucleases for genome engineering in plants: prospects for a bright future. Plant J. 78, 727–741. 10.1111/tpj.12338 24112784

[B45] Raitskin O. Schudoma C. West A. Patron N. J. (2019). Comparison of efficiency and specificity of CRISPR-associated (Cas) nucleases in plants: an expanded toolkit for precision genome engineering. PLoS ONE 14:e0211598. 10.1371/journal.pone.0211598 30811422 PMC6392405

[B46] Ricci C. G. Chen J. S. Miao Y. Jinek M. Doudna J. A. McCammon J. A.. (2019). Deciphering off-target effects in CRISPR-Cas9 through accelerated molecular dynamics. ACS Central Sci. 5, 651–662. 10.1021/acscentsci.9b00020 31041385 PMC6487449

[B47] Russo M. T. Aiese Cigliano R. Sanseverino W. Ferrante M. I. (2018). Assessment of genomic changes in a CRISPR/Cas9 *Phaeodactylum tricornutum* mutant through whole genome resequencing. PeerJ. 6:e5507. 10.7717/peerj.5507 30310734 PMC6174884

[B48] SAS Institute (2018). JMP® 14 Fitting Linear Models, Chu ban: Cary, NC: SAS Institute Inc.

[B49] Scientific Advice Mechanism (SAM) (2017). New Techniques in Agricultural Biotechnology. High Level Group of Scientific Advisors. Available online at: https://ec.europa.eu/research/sam/index.cfm?pg=agribiotechnology# (accessed July 18, 2017).

[B50] Sprink T. Eriksson D. Schiemann J. Hartung F. (2016). Regulatory hurdles for genome editing: process- vs. product-based approaches in different regulatory contexts. Plant Cell Rep. 35, 1493–1506. 10.1007/s00299-016-1990-2 27142995 PMC4903111

[B51] Stemmer M. Thumberger T. Del Sol Keyer M. Wittbrodt J. Mateo J. L. (2015). CCTop: an intuitive, flexible and reliable CRISPR/Cas9 target prediction tool. PLoS ONE 10:e0124633. 10.1371/journal.pone.0124633 25909470 PMC4409221

[B52] Svitashev S. Schwartz C. Lenderts B. Young J. K. Mark Cigan A. (2016). Genome editing in maize directed by CRISPR-Cas9 ribonucleoprotein complexes. Nat. Commun. 7, 1–7. 10.1038/ncomms13274 27848933 PMC5116081

[B53] Tycko J. Myer V. E. Hsu P. D. (2016). Methods for optimizing CRISPR-Cas9 genome editing specificity. Mol. Cell. 63, 355–370. 10.1016/j.molcel.2016.07.004 27494557 PMC4976696

[B54] Walsh S. (2016). Binary logistic regression – what, when, and how, in JMP Discovery Conference 2016 (SAS Institute). Available online at: https://community.jmp.com/t5/Discovery-Summit-2016/Binary-Logistic-Regression-What-When-and-How/ta-p/23902 (accessed November 6, 2020).

[B55] Wang Y. Cheng X. Shan Q. Zhang Y. Liu J. Gao C.. (2014). Simultaneous editing of three homoeoalleles in hexaploid bread wheat confers heritable resistance to powdery mildew. Nat. Biotechnol. 32, 947–951. 10.1038/nbt.2969 25038773

[B56] Woo J. W. Kim J. Kwon S. I. Corvalán C. Cho S. W. Kim H.. (2015). DNA-free genome editing in plants with preassembled CRISPR-Cas9 ribonucleoproteins. Nat. Biotechnol. 33, 1162–1164. 10.1038/nbt.3389 26479191

[B57] Wu X. Kriz A. J. Sharp P. A. (2014). Target specificity of the CRISPR-Cas9 system. Quant. Biol. 2, 59–70. 10.1007/s40484-014-0030-x 25722925 PMC4338555

[B58] Yu Q.-H. Wang B. Li N. Tang Y. Yang S. Yang T.. (2017). CRISPR/Cas9-induced targeted mutagenesis and gene replacement to generate long-shelf life tomato lines. Sci. Rep. 7:818. 10.1038/s41598-017-12262-1 28928381 PMC5605656

[B59] Zhang D. Zhang H. Li T. Chen K. Qiu J.-L. Gao C. (2017). Perfectly matched 20-nucleotide guide RNA sequences enable robust genome editing using high-fidelity SpCas9 nucleases. Genome Biol. 18:191. 10.1186/s13059-017-1325-9 29020979 PMC5637269

[B60] Zhang Y. Liang Z. Zong Y. Wang Y. Liu J. Chen K. (2016). Efficient and transgene-free genome editing in wheat through transient expression of CRISPR/Cas9 DNA or RNA. Nat. Commun. 7, 1–8. 10.1038/ncomms12617 PMC5007326 27558837

[B61] Zhao H. Wolt J. D. (2017). Risk associated with off-target plant genome editing and methods for its limitation. Emerg. Top. Life Sci. 1, 231–240. 10.1042/ETLS20170037 PMC7288994 33525760

[B62] Zhu C. Bortesi L. Baysal C. Twyman R. M. Fischer R. Capell T.. (2017). Characteristics of genome editing mutations in cereal crops. Trends Plant Sci. 22, 38–52. 10.1016/j.tplants.2016.08.009 27645899

[B63] Zischewski J. Fischer R. Bortesi L. (2017). Detection of on-target and off-target mutations generated by CRISPR/Cas9 and other sequence-specific nucleases. Biotechnol. Adv. 35, 95–104. 10.1016/j.biotechadv.2016.12.003 28011075

