# Supplementary material for: Which Factors Affect the Occurrence of Off-Target Effects Caused by the Use of CRISPR/Cas: A Systematic Review in Plants
Source: Front Plant Sci. 2020 Nov 23;11:574959. doi: 10.3389/fpls.2020.574959 (PMC7719684; doi:10.3389/fpls.2020.574959)
Supplement: Supplementary file 1 [file Table_1.DOCX]

|  | TS=((crop OR crops OR plant OR plants OR seed OR seeds OR Arabidopsis OR Tobacco OR Nicotiana OR “green algae” OR “Chlamydomonas reinhardtii” OR hemp OR "Cannabis sativa" OR cereal OR cereals OR corn OR “zea mays” OR maize OR canola OR rapeseed OR oilseed OR Brassica OR wheat OR Triticum OR barley OR hordeum OR oat OR avena OR rye OR "secale cereale" OR rice OR oryza OR soybean OR "Glycine max" OR sorghum OR potato OR Solanum OR "sugar beet" OR "sugar-beet" OR "fodder beet" OR "beta vulgaris" OR alfalfa OR Medicago OR tomato OR cucumber OR cucumis OR carrot OR "Daucus carota" OR pepper OR capsicum OR zucchini OR cucurbita OR "eggplant" OR spinach OR "Spinacia oleracea" OR onion OR allium OR bean OR beans OR phaseolus OR pea OR "Pisum sativum" OR sunflower OR "Helianthus annuus" OR mushroom OR fungi OR beet OR cabbage OR lettuce OR lactuca OR pumpkin OR lentil OR "Lens culinaris" OR leek OR rhubarb OR "Rheum rhabarbarum" OR celery OR "Apium graveolens" OR melon OR lupine OR lupines OR radish OR raphanus OR “fruit tree” OR “fruit trees” OR apple OR apples OR malus OR orange OR "Citrus sinensis" OR banana OR musa OR grape OR grapevine OR vitis OR pear OR Pyrus OR cherry OR "Prunus avium" OR raspberry OR "Rubus idaeus" OR strawberry OR Fragaria OR grapefruit OR "Citrus aurantium" OR lemon OR "Citrus limon" OR lime OR "Citrus latifolia" OR kiwi OR "Actinidia deliciosa" OR garlic OR ginger OR curcuma OR "Piper nigrum" OR parsley OR "Petroselinum crispum" OR peppermint OR "Mentha piperita" OR “sweet basil” OR basilicum OR oregano OR Origanum OR rosemary OR Rosmarinus OR poplar OR manihot OR "Manihot esculenta" OR sugarcane OR "Saccharum officinarum" OR coffee OR Coffea OR cotton OR "Gossypium hirsutum" OR flax OR "Linum usitatissimum" OR dandelion OR Taraxacum OR Peanut OR Peanuts OR "Arachis hypogaea" OR Olive OR "Olea europaea" OR sesame OR "Sesamum indicum" OR cocoa "Theobroma cacao" OR hazelnut OR "Corylus avellana" OR walnut OR "Juglans regia" OR coconut OR "Cocos nucifera" OR jujube OR "Ziziphus jujuba Meikl"OR “camelina sativa”) AND ("genome edit*" OR "genome-edit*" OR "new breeding techn*" OR "new-breeding techn*" OR "Molecular scissors" OR "Designer nucleas*" OR "Site Directed Nucleas*" OR "Site-Directed Nucleas*" OR “site directed mutation*” OR “site-directed mutation*” OR "gene edit*" OR CRISPR* OR "clustered regulatory interspaced short palindromic repeats" OR “clustered regularly interspaced short palindromic repeat*” OR crRNA OR cas9 OR cpf1))  Timespan=2018-2019  Search language=Auto  Databases= WOS, BIOABS, BCI, BIOSIS, CABI, DRCI, DIIDW, KJD, RSCI, SCIELO, ZOOREC |
| --- | --- |
